# Large intra-chromosome heterogeneity leads to emergent nonlinear mechanics

**DOI:** 10.1038/s41567-026-03407-z

**Published:** 2026-08-07

**Authors:** Constantijn van der Smagt, Janni Harju, Tianlong Man, Andreas S. Biebricher, Tinka V. M. Clement, Iddo Heller, Chase P. Broedersz, Gijs J. L. Wuite, Erwin J. G. Peterman

**Affiliations:** 1 Department of Physics and Astronomy and LaserLaB Amsterdam, Vrije Universiteit Amsterdam, Amsterdam, The Netherlands; 2 School of Physics and Optoelectronic Engineering, Beijing University of Technology, Beijing, China; 3 Present Address: Joseph Henry Laboratories of Physics, Princeton University, Princeton, NJ USA

**Keywords:** Biological fluorescence, Biological physics, Optical manipulation and tweezers, Super-resolution microscopy, Single-molecule biophysics

## Abstract

Faithful chromosome segregation during mitosis relies on the formation of compact, individualized chromosomes that withstand drag and spindle-generated forces. Structural failure of mitotic chromosomes under force can disrupt the distribution of genetic material to daughter cells, causing aneuploidy or cancer. The overall mechanical properties of mitotic chromosomes have been suggested to arise from their structural heterogeneity. The magnitude and scale of this heterogeneity have not been measured, leaving its impact on chromosome mechanics unresolved. Here we show that chromosomes are highly mechanically heterogeneous: within one chromosome, the local stiffness can vary by up to two orders of magnitude. This extreme mechanical heterogeneity is exemplified by the centromere, which is an order of magnitude softer than the whole chromosome. These results demonstrate how the mechanical complexity of mitotic chromosomes gives rise to their emergent nonlinear mechanical behaviour, distinct from the polymer properties of their constituents. More broadly, we discuss how structural heterogeneity can shape the nonlinear responses of composite materials, with implications for both understanding biological assemblies and designing new synthetic materials.

## Abstract (web summary)

Analysis of the mechanical properties of mitotic chromosomes is key for understanding the robustness of chromosomes during cell division. It is now shown that chromosomes are highly mechanically heterogeneous.

## Main

For reliable separation during cell division, mitotic chromosomes must form a compact and individualized structure that resists drag forces and perforation by the mitotic spindle^1–3^. Failure to do so may result in the erroneous distribution of genetic material to daughter cells, and the resulting aneuploid (containing an abnormal number of chromosomes) cells may form tumours^4,5^. In interphase, the genome is loosely organized in the cell nucleus, and individual chromosomes cannot be distinguished. On entering mitosis, the concurrent compaction of chromatin through histone deacetylation^3^ and extrusion of chromatin into nested loops by protein complexes condensin I and condensin II (refs. ^6–8^) result in rod-shaped chromatids. Just before their separation in anaphase, sister chromatids are tethered together at their centromeres by the protein complex cohesin and, together, the chromatids form the iconic chromosomal X shape.

Mitotic chromosomes are highly heterogeneous structures. The most striking examples of this heterogeneity are distinct chromatin structures like centromeres and telomeres. These structures differ from the chromosome bulk in their DNA sequence, protein content^9,10^, histone modifications^11–13^ and three-dimensional (3D) chromatin organization^10,14–16^, facilitating their specific functions: the attachment of the mitotic spindle at the centromeres and the capping of the telomeric ends. Additional heterogeneity results from the chromosome’s alternating bands of euchromatin and heterochromatin, corresponding to transcriptionally active and inactive interphase regions^17^ and differing in their histone modifications. Apart from distinct functions, these structures may also exhibit distinct mechanical properties, which could have pronounced effects on the mechanical properties of the chromosome as a whole. However, neither the magnitude nor the scale of intra-chromosomal heterogeneity has been experimentally determined, and the question of its effects on chromosome mechanics remains unanswered. Here we set out to answer this question by spatially resolving the mechanical response in mitotic chromosomes.

## Results

### Combining optical tweezers and 3D stimulated emission depletion

**Fig. 1:**
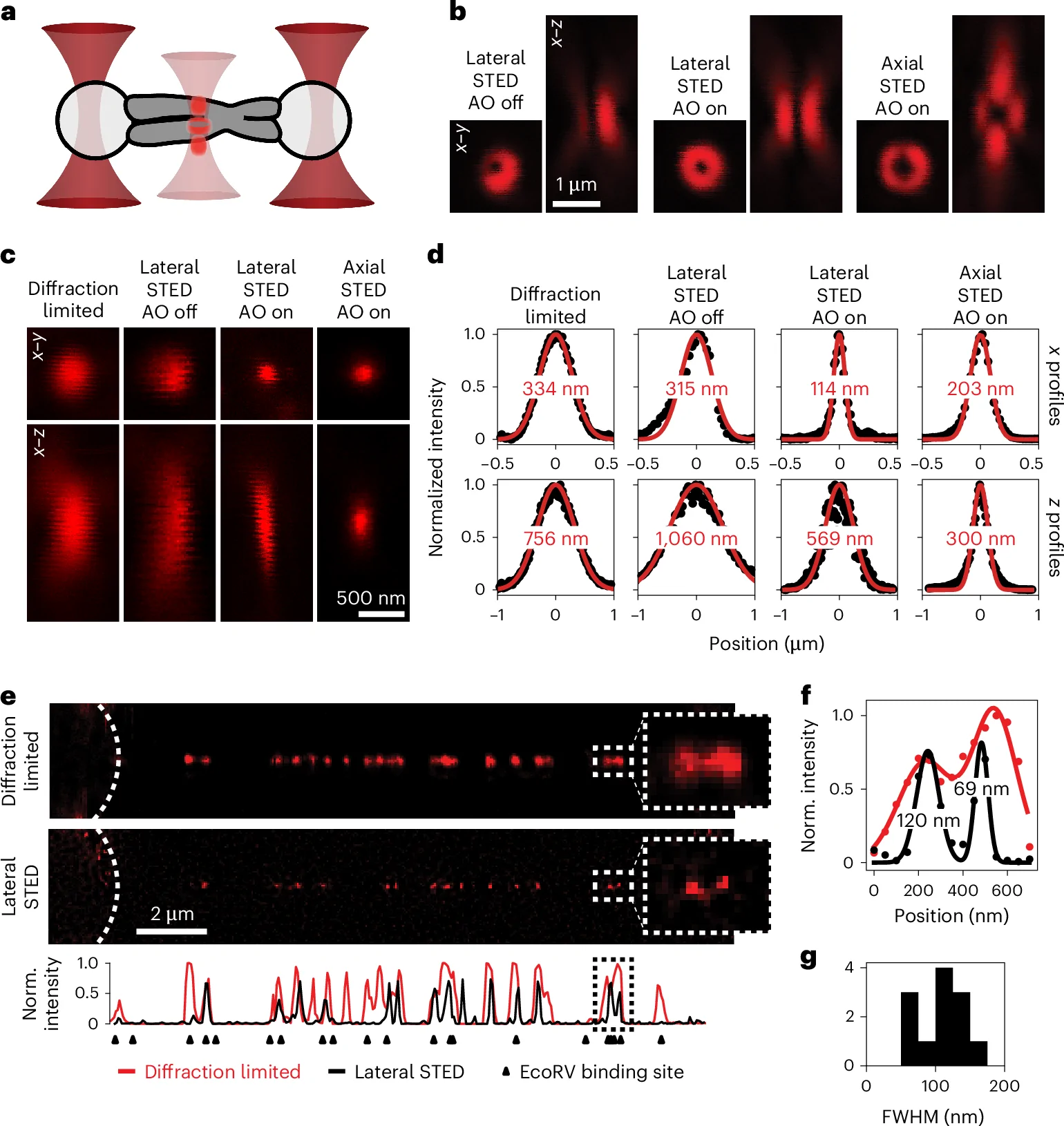
STED allows super-resolution imaging in optical tweezers. **a**, Schematic of optical tweezers and 3D-STED super-resolution experimental setup. **b**, Point spread functions of the STED beam, imaged by reflecting it off 50-nm gold nanoparticles in an agarose gel. **c**, Fluorescence point spread functions for diffraction-limited confocal imaging, lateral STED imaging with/without AO and axial STED imaging with AO. Imaged using 20-nm crimson beads in an agarose gel (Methods). The brightness is normalized. Representative images in **b** and **c** were highly reproducible. **d**, Profiles corresponding to the images in **c**. The FWHMs of the fitted Gaussians are indicated in the figures in red. **e**, Confocal and lateral STED (after deconvolution) images of multiple EcoRV-Atto647N restriction enzymes binding to λ-DNA. Bottom: intensity profile along the λ-DNA, normalized to the highest confocal value, compared with known EcoRV binding sites. Inset: zoomed-in view. The experiment was repeated several times during the validation of the instrument, yielding similar results. **f**, Normalized intensity profiles for the molecules in the inset in **e**. The FWHMs of the STED peaks are indicated in black. **g**, Distribution of the fitted FWHM values for the lateral STED loci in **e**.

**Extended Data Fig. 1:**
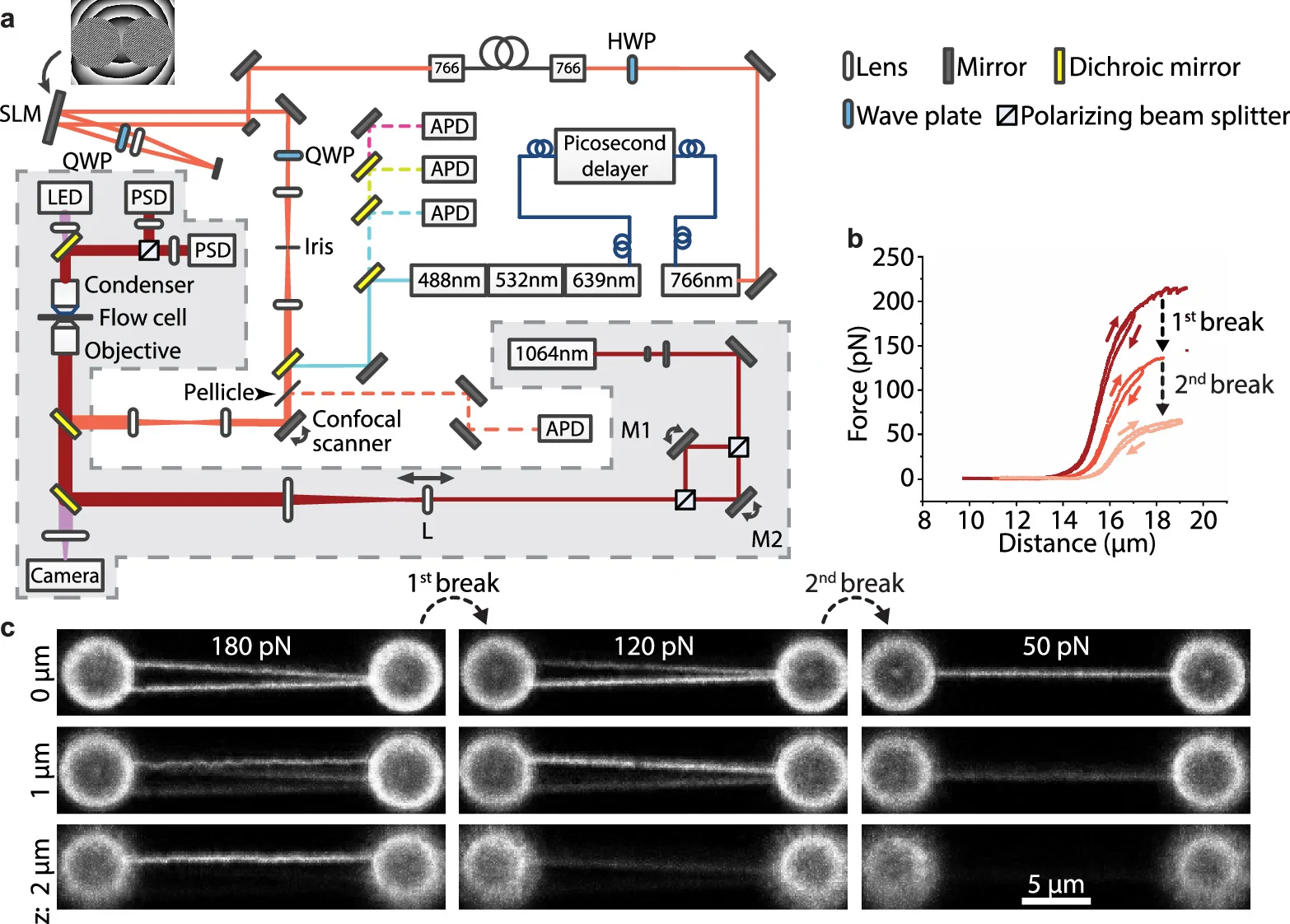
Optical setup and optical tweezers verification. **a**: Schematic for our combined optical tweezers and STED setup. The optical tweezers pathway is indicated in dark red, the STED pathway in light red, and the fluorescence pathway in cyan. Everything inside the gray dashed line was provided by LUMICKS B.V.; everything outside the gray dashed line was homebuilt. See Methods for more details. **b**: Force-distance curves for the stretching of 3 lambda-DNA molecules. The overstretching plateau lowers with each molecule that breaks. The plateaus are at the expected forces. **c**: Diffraction-limited confocal images for the molecules represented in B, taken in 3 different z-planes. The beads can be moved in the z-direction using lens L in S1A. We could calibrate the z-height by making a z-scan of a trapped bead of known size. The experiment was repeated several times during validation of the instrument, yielding similar results.

**Extended Data Fig. 2:**
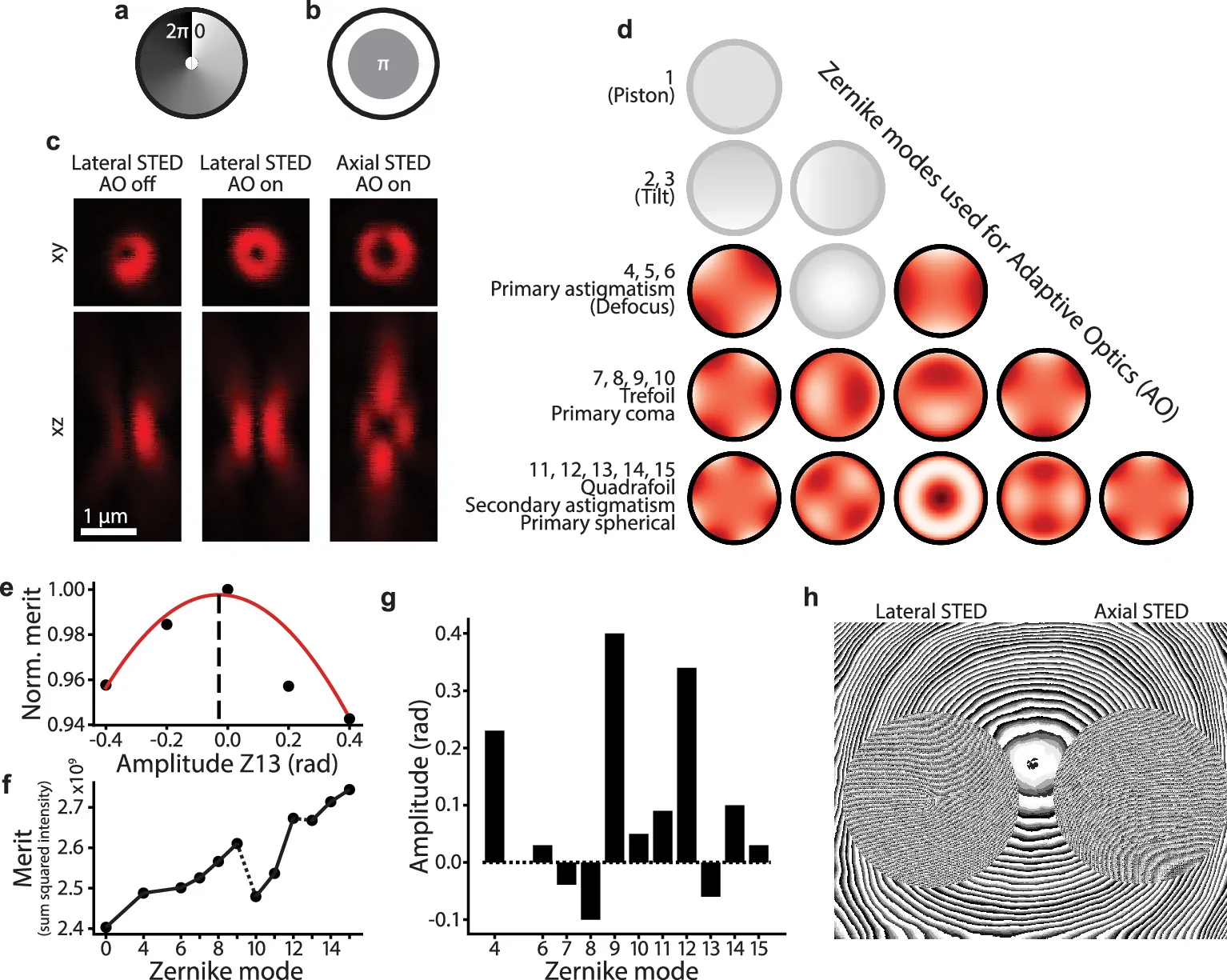
Adaptive optics for STED. **a**: “Helix” phase mask. **b**: “Annular” phase mask. **c**: Point spread functions of the STED beam, imaged by reflecting it off 50 nm gold nanoparticles in an agarose gel. Representative images were highly reproducible. **d**: The first 15 Zernike modes, which we used for adaptive optics. Modes 1, 2, 3, and 5 were not used. **e**: Merit corresponding to a range of amplitudes for Zernike mode 13 (primary spherical). The optimal value for our system was determined to be −0.06 rad. **f**: Evolution of merit (sum of squared pixel intensities) during the adaptive optics procedure. **g**: Optimal Zernike mode amplitudes for aberration correction as determined by the adaptive optics procedure. **h**: Example of a phase mask that is displayed on the SLM.

Studying the spatial mechanical heterogeneity of a mitotic chromosome requires the concurrent precise application of forces to the whole chromosome and the high-resolution imaging of its local deformations. To this end, we developed a novel approach that combines dual-trap optical tweezers^18–20^—to pull on tethered mitotic chromosomes (as described previously^21,22^), with 3D stimulated emission depletion (STED)^23–25^ super-resolution fluorescence microscopy—to monitor the nanoscale deformations of fluorescently labelled chromosome components or segments (Fig. 1a). Accordingly, we extended a LUMICKS optical tweezers instrument with our custom-built STED pathway (Methods and Extended Data Fig. 1). First, we verified the proper functioning of the optical tweezers and diffraction-limited confocal fluorescence microscope (Extended Data Fig. 1). Then, we used a spatial light modulator (SLM) in the depletion-beam pathway to generate the STED doughnut (Fig. 1b), and further optimized the imaging resolution using an adaptive optics (AO) aberration correction protocol^26–30^ (Methods, Supplementary Note 1 and Extended Data Fig. 2). AO optimization resulted in substantially improved lateral and axial resolutions of around 100 nm and 300 nm, respectively (Fig. 1c,d). In the remainder of this work, we used lateral STED, because its improved *x*–*y* resolution turned out to be more valuable to our experiments than the improved *z* resolution achieved by axial STED.

To test our instrument, we monitored the binding of Atto647N-labelled EcoRV—a restriction enzyme—to tethered λ-DNA. EcoRV cleaves a specific short DNA sequence on double-stranded DNA. In the absence of Mg^2+^ and the presence of Ca^2+^, it has been shown to specifically bind but not cleave DNA^31,32^. We used both diffraction-limited confocal and lateral STED microscopy techniques to image the bound restriction enzymes on the DNA (Fig. 1e). Comparing the fluorescence profile along the DNA to the known locations of EcoRV binding sites^33^, we found that most binding sites were covered (18/21), whereas we also identified the binding of EcoRV to six non-specific sites. Between the collection of the confocal image and the subsequent STED image, some molecules bleached or detached from the DNA (3/18 specific, 3/6 non-specific). Restriction enzymes that were close together were difficult to resolve using confocal imaging, but could be resolved using STED microscopy (Fig. 1e (inset) and Fig. 1f), with an average full-width at half-maximum (FWHM) of the STED spots of 120 ± 35 nm (Fig. 1g). We concluded that the combination of optical tweezers and STED imaging functions as expected, with a spatial imaging resolution sufficient for experiments to probe the mechanical heterogeneity of mitotic chromosomes.

### Centromeres are exceptionally soft

**Fig. 2:**
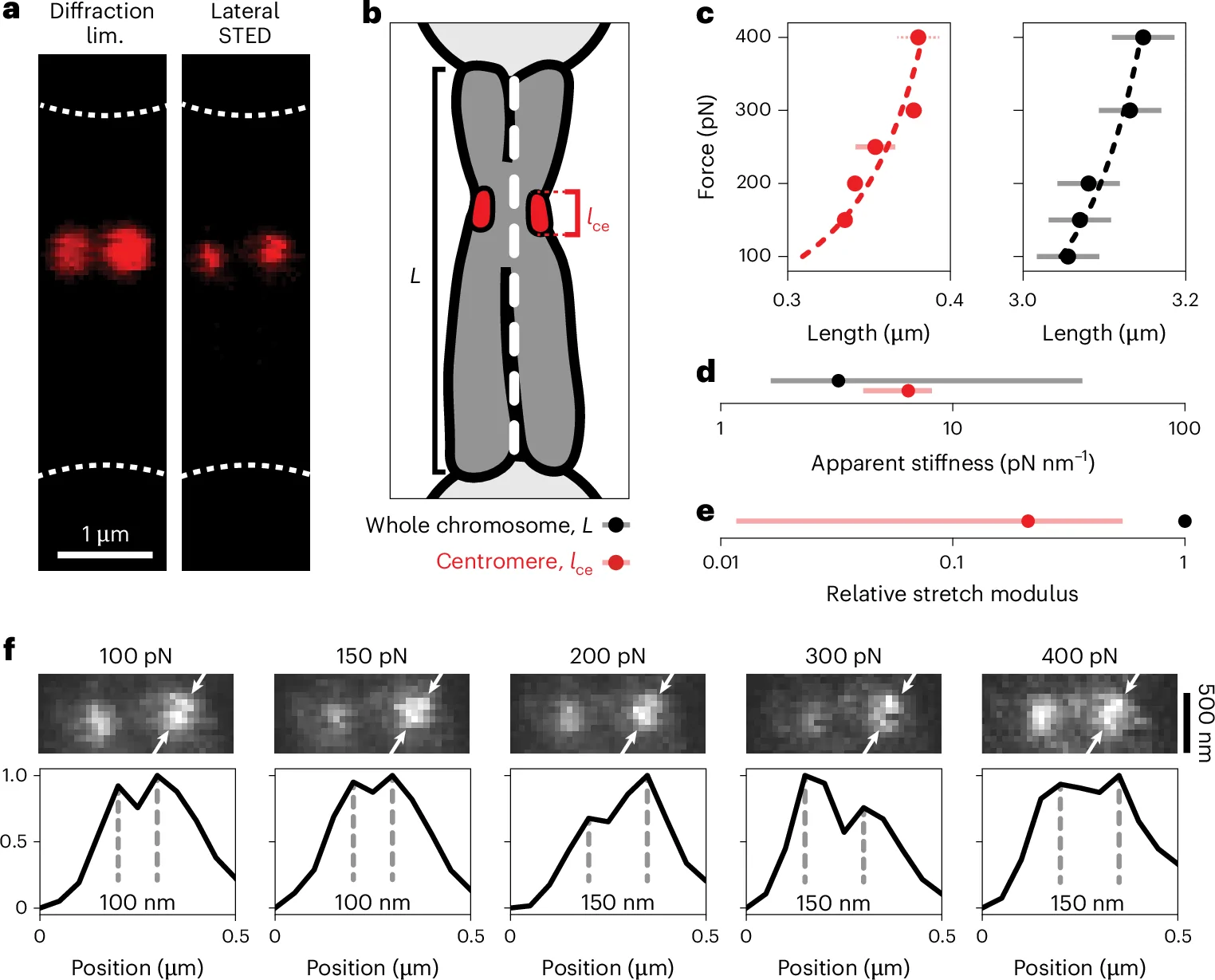
STED imaging of centromeres reveals their softness. **a**, Diffraction-limited (left) and lateral STED super-resolution (right) image of a trapped chromosome with labelled centromeres. Immunofluorescently labelled CENP-A in red. **b**, Schematic of a trapped chromosome with fluorescently labelled centromeres. *L*, the whole chromosome length; *l*_ce_, the length of the centromere along the chromosome axis. **c**, Left: force–extension curves for the centromere imaged in **a**. To generate the error bars, we calculated the standard error of the mean of a dataset consisting of several images of both centromeres over several stretching cycles. The number of data points for the centromere length varies per force value between *n* = 2 and *n* = 6 (150 pN, *n* = 2; 200 pN, *n* = 6; 250 pN, *n* = 4; 300 pN, *n* = 4; 400 pN, *n* = 2 (technical replicates). For *n* = 2, the error bar is indicated by a dotted line because there the standard error of the mean is not accurately defined. The dashed line shows the curve that was fitted using the generalized WLC model (Methods). Right: force–extension curves for the chromosome imaged in **a**. For error bars, see Methods. **d**, Apparent stiffness for the whole chromosome and for the centromere in **a** and **c** (dots), calculated using the chromosome and centromere lengths at 150 pN and 400 pN. For the calculation of the error bars (lines), see Methods. **e**, Normalization of the data in **d**. For the calculation of the error bars (lines), see Methods. **f**, Zoomed-in views of the lateral STED super-resolution images of the centromeres at the indicated pulling force. A bipartite structure can be observed in the right centromere. The two parts of the bipartite structure can be resolved satisfying the Rayleigh criterion in the image taken at 300 pN, and Dawes’s limit at 100 pN and 150 pN. Axial drift and random noise prevent the strict quantitative resolution of both centromere domains in the images taken at 200 pN and 400 pN. The white arrows indicate the axis that was used to calculate the normalized intensity profiles that are plotted at the bottom. The distance between peaks is indicated. The pixel size is 50 nm. In our experiments, a clear bipartite centromere was observed once.

We started our study by focusing on centromeres—the chromosome regions that connect the sister chromatids and attach to the mitotic spindle. Centromeres diverge from other parts of the chromosome in terms of DNA sequence^34,35^, histone variants^35–38^ and chromatin organization^10,36,38^, which makes them a compelling starting point for probing mechanical heterogeneity in chromosomes. To probe centromere stiffness, we first immunostained isolated human metaphase chromosomes for centromeric histone variant CENP-A (Methods). Then, we tethered these chromosomes between optically trapped beads as described previously^21,22^ (Fig. 2a), and imposed force clamps at forces ranging from 100 to 400 pN (Methods). We imaged each chromosome at regular force intervals, determining the length *L* of the whole chromosome from the autofluorescence of the chromosome body and polystyrene beads, and the centromere length *l*_ce_ from the super-resolution fluorescence images (Fig. 2b), resulting in force–extension curves for the whole chromosome as well as the centromere (Fig. 2c).

To assess how the stiffness of the centromeres compares with that of the whole chromosome, we first calculated their ‘apparent stiffness’ $\Delta F/\Delta {l}_{{\rm{ce}}}$ (Fig. 2d), defined as the total change in force F divided by their deformation (along the direction of the pulling force). The apparent stiffness is defined in absolute terms, and thus, it cannot be used to meaningfully compare chromosome compartments of different sizes. To facilitate comparison, we normalized the apparent stiffness by calculating the dimensionless relative stretch modulus as

1 $${\mu }_{{\rm{rel}}}=\frac{\Delta F}{\Delta {l}_{{\rm{ce}}}}{l}_{{\rm{ce}}}({F}_{\min })/\left(\frac{\Delta F}{\Delta L}L({F}_{\min })\right),$$

where *L* and ΔL are the length and deformation of the whole chromosome, and ${F}_{\min }$ is the lowest force at which the data were collected. The relative stretch modulus of any segment of a homogeneous material will always be equal to 1. Deviations from 1 only occur in the presence of mechanical heterogeneity, where ${\mu }_{{\rm{rel}}} < 1$ indicates that the system is locally softer than the whole chromosome, whereas ${\mu }_{{\rm{rel}}} > 1$ indicates that the system is locally stiffer (Methods). Remarkably, using this normalization, we found that centromeres are almost an order of magnitude softer than the whole chromosome (Fig. 2e).

To investigate the origins of centromere softness, we inspected our super-resolution images and found two centromere domains separated by a distance of around 100–150 nm (Fig. 2a,f). This is consistent with recent experiments^10^ showing that centromeres in vertebrate mitotic chromosomes consist of a ‘bipartite structure’, in which the two halves preferentially interact with their respective associated chromosome arm. In our experiments, the separation between centromere domains seems to increase with the applied force (Fig. 2f), suggesting that the centromere’s bipartite structure might be an important cause for its relative softness. To conclude, the centromeres constitute a striking example of mechanical heterogeneity caused by local structural, genetic and epigenetic variations.

### Chromosomes are highly heterogeneous

**Fig. 3:**
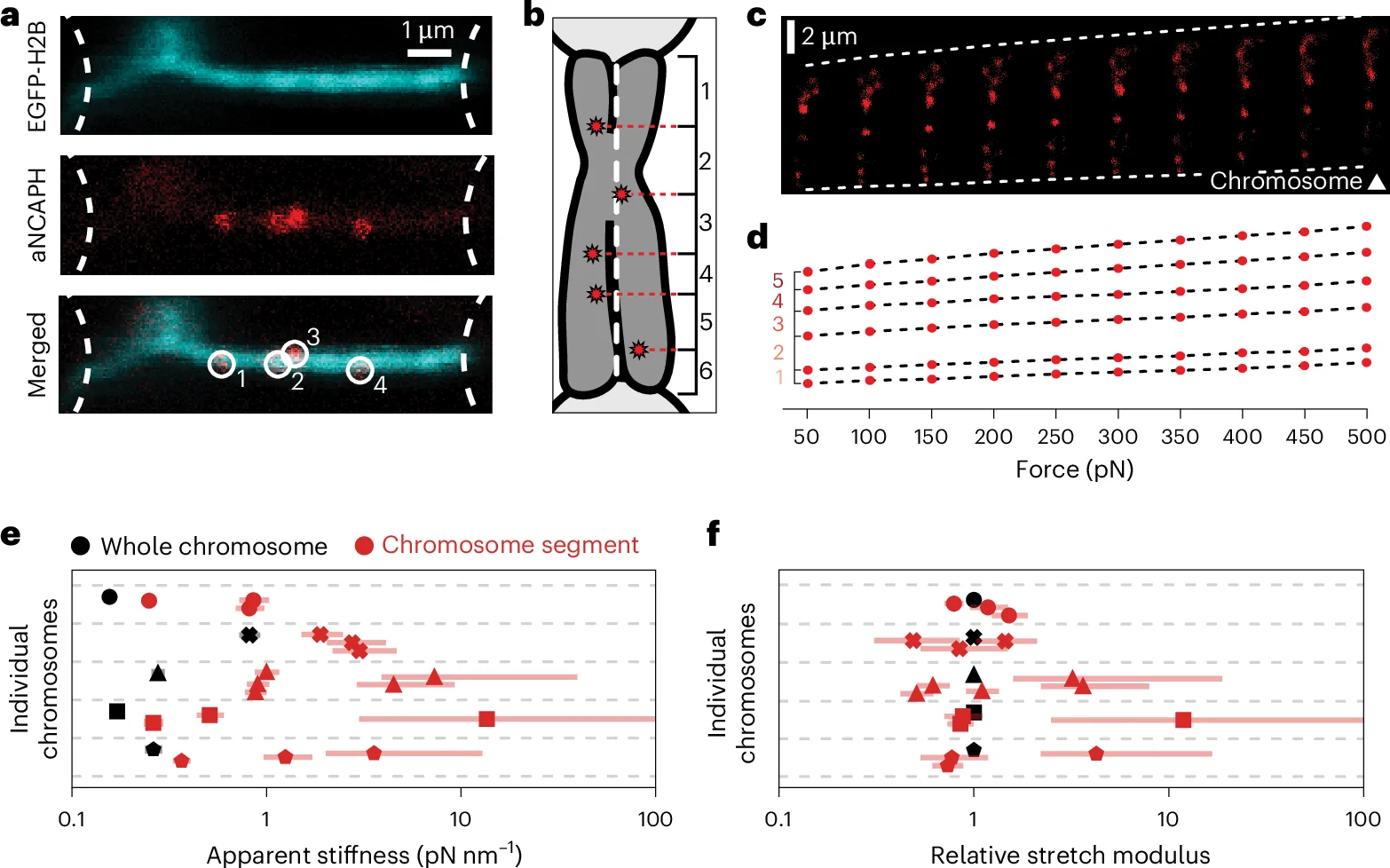
Chromosome segments show mechanical heterogeneity. **a**, Sparsely immunostained chromosome in an optical tweezers experiment, clamped at 50 pN, and imaged using diffraction-limited confocal microscopy. The chromosome is indicated by the black circle in **e** and **f** and red circles in Fig. 4f. Spots 1 and 3 bleached prematurely and were not used, leaving three segments. Similar sparse staining of trapped chromosomes was achieved repeatedly. **b**, Schematic of the visual segmentation of a randomly sparsely immunostained chromosome. **c**, Sparsely immunostained chromosome in an optical tweezers experiment, clamped over forces ranging from 50 to 500 pN and imaged using regular confocal microscopy. Similar experiments were successfully (without premature bleaching or disintegration of the sample) completed five times (**e** and **f**). **d**, Traces extracted from the chromosome in **c**. Traces correspond to the chromosome indicated by the black triangle in **e** and **f** and red triangles in Fig. 4f. **e**, Apparent stiffness for the whole chromosome and per segment for five different chromosomes. Each chromosome is indicated using a different shape. Chromosome samples were derived from two separate isolations from the same cell line. **f**, Normalization of the data in **e**. For the calculation of the error bars (lines) in **e** and **f**, Methods see.

**Extended Data Fig. 3:**
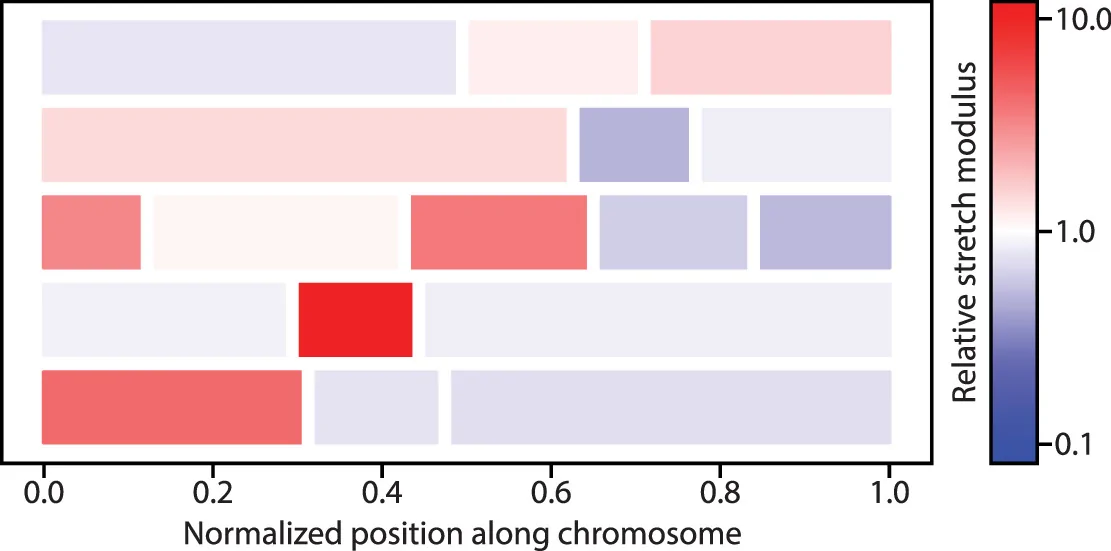
Relating relative stretch modulus to position along chromosome. Segments are plotted for the 5 chromosomes from Fig. 3f. The segments are normalized to the whole chromosome length. Their relative stretch modulus is indicated by the color, as defined by legend on the right.

To test for further spatial heterogeneities outside of the centromeric region, we set out to measure the force–extension profiles of individual segments along the chromosome length. To generate random, visually discernable chromosome segments, we immunostained for NCAPH (a component of the condensin I protein complex; Methods), which is distributed roughly uniformly throughout chromosomes^7^. Low concentrations and short incubation times resulted in a sparse and randomly distributed antibody coverage along the chromosome (Fig. 3a,b). We force clamped the stained chromosomes at regularly spaced forces ranging from 50 to 500 pN, taking fluorescence images at each applied force (Fig. 3c). In the sparse-immunostaining regime, fluorescence spots were rarely overlapping, rendering the use of STED super-resolution imaging superfluous; therefore, we refrained from using STED in some cases to limit photobleaching. For all force sweeps, we pinpointed loci that were visible in all image frames and defined them, as well as both bead edges, as segment borders (Fig. 3d). To assess whether the segments vary in stiffness, we calculated their apparent stiffnesses $\Delta F/\Delta l$ (Fig. 3e and Methods) and, subsequently, determined their relative stretch moduli following the procedure outlined before (equation (1), Fig. 3f and Methods). Remarkably, we encountered a broad range of mechanical behaviours, including moduli of up to ten times that of the whole chromosome. We did not find abnormalities in the relative stretch moduli of the segments that were attached to the beads, or any correlation between the positions of the segments and their relative stretch moduli in general (Extended Data Fig. 3). Including the discussed data on the centromere, the found relative stretch moduli range over two orders of magnitude, demonstrating that mitotic chromosomes exhibit substantial mechanical heterogeneity along their long axis.

### Emergent nonlinearity in complex materials

Previous optical tweezers experiments have demonstrated that human mitotic chromosomes exhibit an anomalous nonlinear force response^21,39^, which may depend on the loading rate^39^. The chromosome stiffness, $K\left(F\right)=\frac{{\mathrm{d}}{F}}{{\mathrm{d}}{L}}$, is constant in the linear regime at low applied force, whereas at high forces, it increases surprisingly gradually according to a power law ($K\propto {F}^{{{\gamma }}}$), with a stiffening exponent ${{\gamma }}\approx 0.85$ (refs. ^21,39^). This value is below the theoretical lower limit for homogeneous polymer models (${{\gamma }}$ > 1), such as the worm-like chain (WLC) model, for which ${{\gamma }}=3/2$. This finding can be explained by the hierarchical worm-like chain (HWLC) model, which describes the chromosome as a composite material consisting of a series of mechanically distinct WLCs^21^. Owing to its composite nature, individual material components do not stiffen all at once, but sequentially. The force-induced sequential stiffening of the heterogeneous WLC components gives rise to an emergent nonlinearity for the system as a whole, with an effective stiffening exponent ${{\gamma }} < 1$, lower than the exponents ${{\gamma }} > 1$ that characterize the system’s microscopic constituents. This model, hence, conceptually explains how structural heterogeneity can cause composite materials to stiffen abnormally gradually.

### Chromosome segments stiffen anomalously

We did not expect the chromosome segments in our experiments to correspond to mechanically homogeneous regions, since their fluorescently demarcated borders were randomly generated. To confirm that the segments themselves exhibit mechanical heterogeneity, we characterized their nonlinear force–extension behaviour. Anomalous stiffening exponents ${{\gamma }} < 1$ would provide evidence for substantial mechanical heterogeneity at length scales below the segmentation length. To determine stiffening exponents by fitting to the force–extension data, we first set out to formulate a generalization—suitable for heterogeneous polymers—of the approximate Marko–Siggia formula for flexible WLCs^40^:

2 $$\frac{F}{{f}_{\mathrm{c}}}\approx \frac{l}{{l}_{\mathrm{c}}}+\frac{1}{4}\left({\left(1-\frac{l}{{l}_{\mathrm{c}}}\right)}^{2}-1\right).\,$$

Here ${f}_{\mathrm{c}}$ is the critical force of the sub-chain and ${l}_{\mathrm{c}}$ is its contour length. The segment stiffness $k\left(F\right)={{\mathrm{d}}{F}}/{{\mathrm{d}}{l}}$ is a constant ${k}_{0}$ in the linear regime, where $l\ll {l}_{\mathrm{c}}$, and then asymptotically follows a power law ($k\sim {F}^{\gamma }$) with γ=3/2 as $l\to {l}_{\mathrm{c}}$. To describe a heterogeneous segment with any stiffening exponent ${{\gamma }} > 1$, we propose a generalized interpolation formula:

3 $$\frac{F}{f_{\mathrm{c}}}\approx \frac{l}{{l}_{\mathrm{c}}}+{B}_{ > }\left({\left(1-\frac{l}{{l}_{\mathrm{c}}}\right)}^{1/\left(1-\gamma \right)}-1\right).$$

Here ${B}_{ > }$ is a positive constant of order unity, set by the scaling coefficient of the linear-response coefficient with ${f}_{\mathrm{c}}$ and ${l}_{\mathrm{c}}$: ${k}_{0}=\frac{{f}_{\mathrm{c}}}{{l}_{\mathrm{c}}}(1+\frac{{B}_{ > }}{{{\gamma }}-1})$. In the limit $l\to {l}_{\mathrm{c}},$ the second term dominates, and differentiation yields the required scaling of $k\propto {F}^{\gamma }$.

For anomalous exponents ($0 < {{\gamma }} < 1$) in systems with emergent nonlinearity, by contrast, the correct asymptotic scalings for the stiffness are captured by

4 $$\frac{F}{{f}_{\mathrm{c}}}\approx {A}_{ < }\frac{l}{{l}^{* }}+{B}_{ < }{\left(\frac{l}{{l}^{* }}\right)}^{1/\left(1-\gamma \right)}.$$

**Fig. 4:**
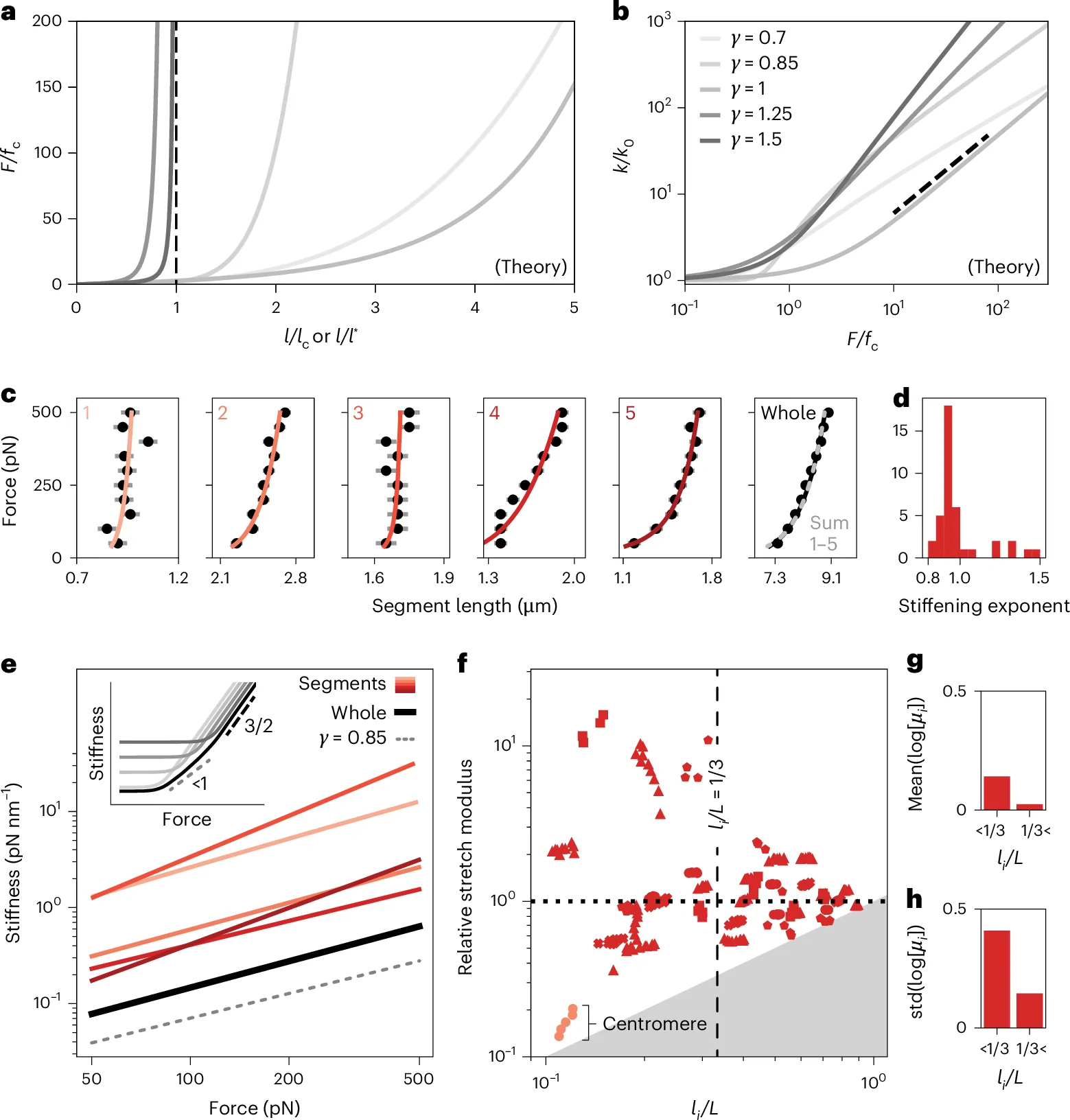
Mechanical heterogeneity is present within chromosome segments. **a**, Theoretical force–extension curves corresponding to different stiffening exponents ${{\gamma }}$, calculated using equations (3), (4) and (9). The legend is shown in **b**. For ${{\gamma }}\le 1$, the *x* axis is normalized with respect to the critical length ${l}^{* }$. For ${{\gamma }} > 1$, the *x* axis is normalized with respect to the contour length ${l}_{\mathrm{c}}$, such that $l/{l}_{\mathrm{c}} < 1$ (vertical dashed line). **b**, Theoretical stiffness–force curves for different values of the stiffening exponent *γ*. The dashed line indicates an exponent of 1. The *y* axis is normalized by the stiffness at the lowest force. **c**, Segment-wise force–extension curves corresponding to the chromosome in Fig. 3c were fitted using the generalized WLC model. Error bars are given by $\sqrt{2}\Delta$ in both directions, where $\sqrt{2}\Delta$ is the localization precision. These segments correspond to the triangles in **f** and Fig. 3e,f. The grey dashed line in the rightmost panel represents the sum of segment fits 1 to 5. Curves correspond to a single experimental instance. **d**, Distribution of stiffening exponent *γ* resulting from fitting to force–extension curves of all chromosome segments and combinations of neighbouring chromosome segments, including full chromosomes. Data were taken from the five chromosomes displayed in Fig. 2e,f. **e**, Stiffness–force curves derived from the fits in **c**. The slope in this log–log plot indicates the stiffening exponent as γ=0.85. The stiffening exponent of full chromosomes was experimentally found to be γ=0.82 (ref. ^21^) and γ=0.88 (ref. ^39^). A length-normalized plot of this chromosome and a few others is shown in Extended Data Fig. 5. Inset: stiffness–force curves for four flexible WLCs, calculated using equation (2), as well as the curve corresponding to their serial HWCL response (black full line). Sequential stiffening occurs in the region where the component curves cross, and the series exhibits an anomalous stiffening exponent (dashed lines). **f**, Relative segment length versus relative stretch modulus. For a perfectly homogeneous chromosome, the latter quantity would always be equal to 1, as indicated by the dotted line. Data points include all chromosome segments and combinations of neighbouring segments, at all forces (number of chromosomes, *n* = 5). Marker shapes correspond to that in Fig. 3. Data for the centromere from Fig. 2 are also included. The grey area indicates the inaccessible region, ${k}_{i}{l}_{i}/(KL) < {l}_{i}/{L}.$ **g**, Mean of the logarithm of the relative stretch modulus, for data from **f**. Statistical testing (two-sided Welch’s *t*-test, *P* = 0.048) suggests a small but significant difference between the means of the two groups. However, in Extended Data Fig. 5, the difference in mean was found to be not significant. **h**, Standard deviation of the logarithm of the relative stretch modulus, for data in **f**. Levene’s test of equal variance (two sided) confirms that the difference in variance between groups is statistically significant (*P* = 8.1 × 10^−14^).

Here ${A}_{ < }$ and ${B}_{ < }$ are positive constants of similar order, and ${k}_{0}={A}_{ < }\frac{{f}_{\mathrm{c}}}{{l}^{* }}$. The contour length is replaced by a critical extension ${l}^{* }$: the chain can exhibit extensions $l > {l}^{* }$. This formula, thus, suggests that the chain would be infinitely extendable. However, for real materials, we expect the scaling to transition to a stiffening exponent ${{\gamma }} > 1$ once all its components have started to stiffen. As long as we probe a force range in which the material exhibits a single stiffening exponent, equations (3) and (4) (and equation (9)) can be used to describe the force–extension curve of a heterogeneous material with any stiffening exponent ${{\gamma }} > 0$ (Fig. 4a,b).

**Extended Data Fig. 4:**
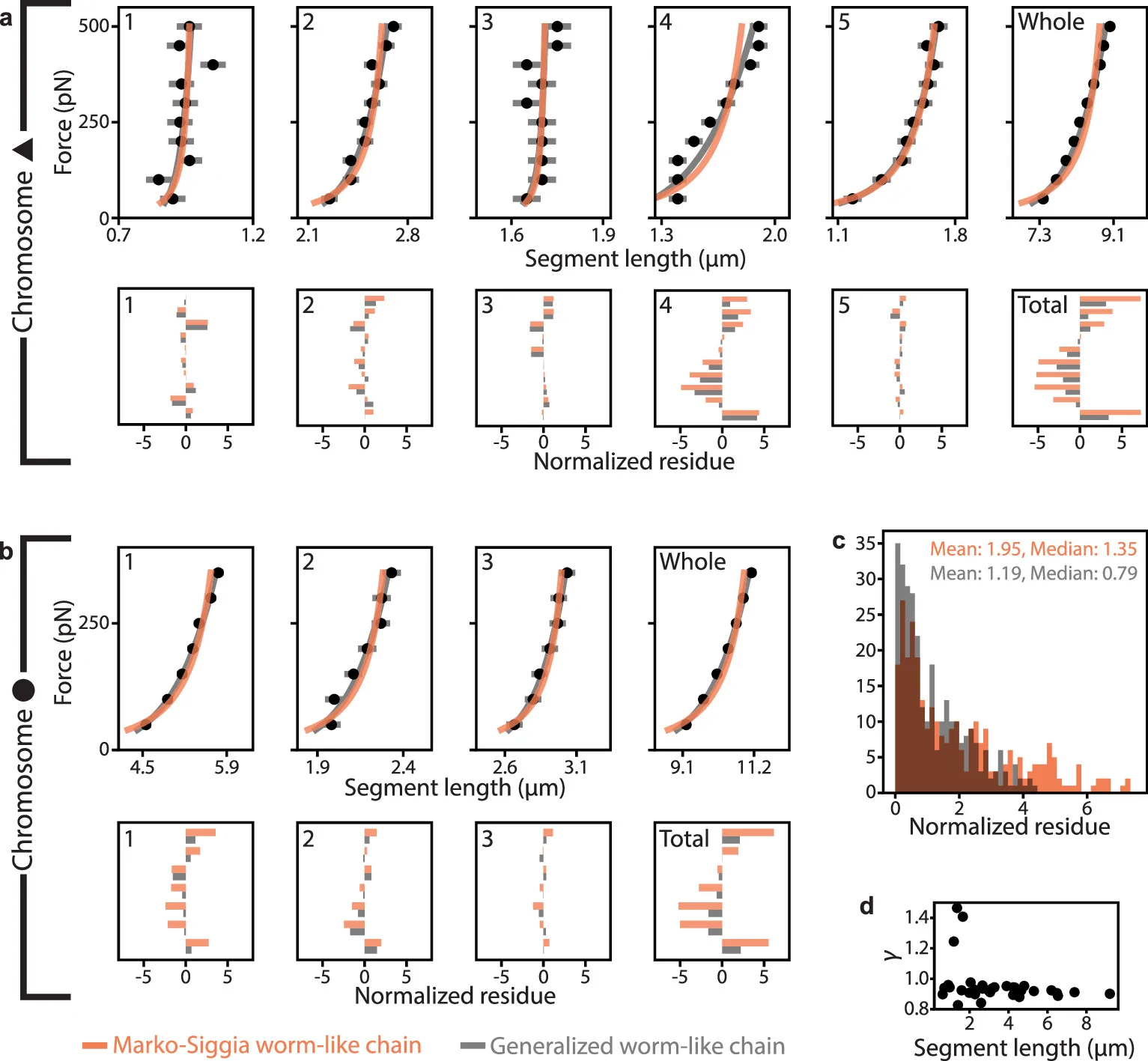
Marko-Siggia WLC fitting vs. generalized WLC fitting. **a** & **b**: The segment-wise force-extension curves of, respectively, the ▲-chromosome and ●-chromosome as in Fig. 3. The data was fitted to the Marko-Siggia WLC formula in orange, and to our generalized WLC formula in gray (Methods). Error bars are given by $\sqrt{2}\Delta$ in both directions, where Δ is the localization precision. Below the curves we plotted the residues of both fits, given in numbers of error bars. For small and stiff segments both formulas fit equally well. For longer segments, that are more HWLC-like, the Marko-Siggia WLC formula tends to underestimate the extension at low and high forces, while it overestimates the extension in the medium force region. The generalized model does better in these cases, by fitting a stiffening exponent γ < 1. Both segment A4 and B2 show some anomalous stretching at low forces that could be due to twisting. By excluding the low force data the remaining data points can be fitted better, but we decided not to, in order not to introduce bias. The neighboring segments seem not affected. For both A and B, curves correspond to a single experimental instance. The ▲-chromosome and ●-chromosome were derived from the same chromosome isolation. **c**: Normalized residues for both fitting models. Data for all segments and combinations of neighboring segments and full chromosomes. For the Marko-Siggia WLC model, 42% of data points are fitted within 1 error bar and 62% within 2 error bars. For the generalized WLC model the numbers are 55% and 79%, respectively. **d**: Segment length versus stiffening exponent γ. One chromosome lacked sufficient low-force data to determine γ accurately and was excluded.

Considering our force–extension data, although some short segments are well described by the conventional Marko–Siggia WLC formula^40^ (equation (2)), the extension curves of whole chromosomes and longer segments are not. For these segments, the stiffening exponent (γ=3/2) is too high, and the extensions at low and high forces are systematically underestimated, whereas extensions in the mid-force range are overestimated (Extended Data Fig. 4). Conversely, by allowing for lower stiffening exponents ($0 < \gamma \le 3/2$), the generalized formulas in equations (3) and (4) give a better fit for all the observed segments (Fig. 4c, Extended Data Fig. 4 and Methods). Most segments are best described by stiffening exponents comparable with the previously found value for whole chromosomes (Fig. 4d; segment median, γ=0.94), indicating that the segments themselves exhibit emergent nonlinearity, and thus, mitotic chromosomes exhibit mechanical heterogeneity even below our average segmentation length scale. Furthermore, the force–extension curve of a whole chromosome can be predicted by combining the force–extension behaviours of its segments (Fig. 4c, last panel), directly demonstrating that the nonlinear stiffening behaviour of the chromosome can be understood as arising from the heterogeneity of its components.

By differentiating the fitted force–extension curves of all chromosome segments, we calculated their stiffness–force curves (Fig. 4e), ${k}_{i}(F)$, where the slope of a curve on a log–log plot corresponds to the stiffening exponent γ. In the HWLC model, all segments consist of WLCs and have the same stiffening exponent (γ=3/2). The WLC elements stiffen sequentially, which can be visualized in a stiffness–force plot as the curves of elements crossing each other (Fig. 4e, inset). Since our current experiments did not probe forces below 50 pN, we could not determine the initial stiffnesses and critical forces of the segments. Furthermore, we have shown that our experimental segments are not WLCs but themselves exhibit sequential stiffening. However, in the specific example of Fig. 4e, the softest segment at low forces stiffens faster than the next softest segment, and the curves cross at ~100 pN. Thus, even in this force range and at this level of segmentation, we directly observed sequential stiffening, providing further proof that the gradual stiffening of mitotic chromosomes reflects emergent nonlinearity that is well captured by the HWLC model.

### Heterogeneity arises at the micrometre scale

To gain deeper insights into the length scale at which the heterogeneity in mitotic chromosome stiffness arises, we analysed how the relative stretch modulus of segment *i*, ${{\mu }_{i}=k}_{i}{l}_{i}/({KL})$, varies with its fractional length ${l}_{i}/L$ (Fig. 4f). Here *K* is the stiffness of the whole chromosome. At each force, we calculated the relative stretch modulus by taking ${l}_{i}$ and *L* directly from the images, and ${k}_{i}$ and *K* from the fitted curves. To extend the range of segment lengths, we considered not just all individual segments but also all combinations of neighbouring segments. In doing so, we observed that large segments, in which spatial heterogeneity is averaged out, have relative stretch moduli close to unity. By contrast, the relative stretch moduli of smaller segments show large deviations (Fig. 4f–h), reflecting increased inter-segment heterogeneity. In particular, we observed several segments that exhibited relative stretch moduli an order of magnitude higher but no segments that exhibited relative stretch moduli an order of magnitude lower than the homogeneous case. This is because ${k}_{i}/K$ is strictly larger than one, resulting in a lower bound for the relative stretch modulus: ${k}_{i}{l}_{i}/({KL}) > {l}_{i}/L$ (Fig. 4f, grey area). At ~1/10 of the whole chromosome length, the centromere is the shortest and also the softest segment we probed, with a relative stretch modulus of ~0.2 (Fig. 4f). The inter-segment variations appear to sharply increase for segment lengths of ≲*L*/3 or on an absolute scale of ≲2 µm (Extended Data Fig. 6). This transition length is much larger than relevant microscopic length scales like those of condensin loops and topoisomerase IIα crosslinking (~10–100 nm (refs. ^7,41,42^)), but is comparable with the macroscopic length scale of chromosome bands and regions such as the centromere (~1 µm). Although large mechanical variability at the length scale of the mesh size is expected in homogeneous polymeric materials^43^, this substantial mechanical heterogeneity at micrometre scales is remarkable.

## Discussion

In this work, we probed the spatial mechanical heterogeneity of mitotic chromosomes by combining optical tweezers and super-resolution immunofluorescence imaging. First, by specifically staining the centromere, we found that centromeres are notably softer than the chromosome arms, which could be related to previous observations that vertebrate centromeres exhibit a bipartite structure during mitosis^10^. The centromere’s softness could be beneficial for its function in several ways. First, a looser organization could facilitate transcription at the centromere during mitosis^44^. Second, the centromere might act as a ‘hinge’ to allow the chromatid to fold as it is pulled through the cytoplasm during anaphase. Finally, a centromere that is softer—not just in the axial but also in the perpendicular direction—might provide a more sensitive sensor for incorrect spindle attachments^45–47^ (Supplementary Note 2), thereby contributing to more accurate chromosome segregation.

Our experiments using sparse labelling have revealed pronounced mechanical heterogeneity along the entire chromosome length, with relative stretch moduli ranging over two orders of magnitude. Apart from the exceptionally soft centromere, another likely source of this mechanical heterogeneity is the alternating bands of transcriptionally active euchromatin and inactive heterochromatin regions, which have been reported to differ in their mechanical properties^48–50^. To investigate the origin of heterogeneity further, our approach could be extended by labelling other chromosomal regions, such as the telomere, heterochromatin, euchromatin or proteins like condensin complexes and the highly abundant topoisomerase IIα, to test whether the stiffness of a chromosome segment correlates with the presence or density of a certain protein or structure.

To conclude, we have shown how the anomalous nonlinear force response of the mitotic chromosome emerges from mechanical heterogeneity among its parts. Therefore, we propose that the mitotic chromosome could be considered an ‘evolved meta-material’, whose large-scale mechanical properties arise from its complex structure and differ from those of the underlying constituents. Mitotic chromosomes are not the only organic materials characterized by such emergent properties; fibrin gels from blood clots^51^, bacterial cell walls^52^, eukaryotic cells^53^ and spider silk^54^ have all been shown to exhibit gradual sequential stiffening, and we expect this to apply to many more biological systems that comprise a hierarchical or composite structure. Our experimental method could be used to test for mechanical heterogeneity and emergent nonlinear properties in other micrometre-scale systems, such as nuclei^55^ or red blood cells^56^. Additionally, the principle of sequential stiffening could be used to design synthetic meta-materials exhibiting desired force–extension responses, with potential applications in sensors, soft robotics and shock absorbers, as well as in the emerging field of physically learning materials^57,58^.

## Methods

### Confocal and STED fluorescence microscopy

For reference, see Extended Data Fig. 1a. A laser system (MCS3 F1, LASOS lasertechnik) that provides three excitation lines (488-nm, 532-nm and 639-nm central-wavelength continuous lasers, and 639-nm central wavelength 20-MHz pulsed laser) was used for confocal and STED fluorescence excitation. The three linearly polarized lasers from the fibre output were collimated using an achromatic Hastings triplet lens (*f* = 40 mm; anti-reflection coating, 400–700 nm; TRH254-040-A-ML, Thorlabs) to get a beam of 7-mm diameter before being converted to circular polarization using a quarter-wave plate (*λ*/4, 400–800 nm, Thorlabs). An excitation filter (FF01-390/482/532/640-25, Semrock) was used to clean up the laser lines. A 766-nm pulsed laser was used for STED (20 MHz, VisIR-765-HP, PicoQuant). The excitation laser was triggered by the STED laser, and a picosecond delayer (PSD-065-A-MOD, Micro Photon Devices) was used to control the timing of the excitation and STED light pulses that arrived on the sample plane. The STED laser was coupled into a polarization-maintaining single-mode fibre (P3-630PM-FC-10, Thorlabs) and subsequently collimated by a 12.5-mm diameter achromatic lens (*f* = 40 mm, near-infrared, Linos) again to get a beam of 7-mm diameter. The length of the fibre was custom-designed to introduce a temporal delay that is larger than the temporal coherence length of the STED laser between the light that is being transmitted along the *s*- and *p*-polarization directions. The polarization orientation of the linearly polarized STED beam was controlled using a half-wave plate (*λ*/2 500–900 nm achromatic, Bernhard Halle) before entering the polarization-maintaining fibre such that the output beam has two incoherently linearly polarized components with orthogonal polarization states. The tuning of the power ratio between these two polarization components, which enables the switching between lateral and axial STED modes, was performed by adjusting the angle of the half-wave plate. The STED beam was then guided to the SLM (X13139-02, 1,280 × 1,024 pixel^2^ with a pixel pitch of 12.5 μm, Hamamatsu). The two polarization components of the beam were then sequentially modulated by the phase masks for lateral and axial STED (displayed on the left and right parts of the screen of the SLM, respectively) using a self-imaging 4*f* telescope system with a quarter-wave plate (AQWP10M-980, Thorlabs). A D-shaped mirror (BBD1-E02, Thorlabs) was used to couple out the STED beam. The two linearly polarized components in the STED beam were converted into two opposed circularly polarized states using a quarter-wave plate (AQWP05M-600, Thorlabs). A 4*f* telescope system (two achromatic lenses with the same focal length, AC254-400-B, Thorlabs) with an iris on the intermediate plane was used to filter out the unmodulated light. The STED and excitation beams were then combined using a dichroic mirror (F48-677, AHF). The combined beams were scanned through the sample using a fast two-dimensional galvo scanning system (Yanus IV digital scan head, TILL Photonics) before being magnified by a 1:4 beam expansion system (two achromatic lenses, AC254-100-AB and AC508-400-A, Thorlabs) and combined with the trapping laser using a dichroic mirror (F43-800, AHF). The back aperture of the objective (Plan Apo IR 60×/1.27 WI, Nikon) was overfilled by the STED and excitation beams. The SLM plane was conjugated with the scanning mirrors plane and then the back aperture plane of the objective lens, to optimize the phase modulation efficiency and cancel any pivoting during the beam steering. An automated *x*–*y* stage (MicroStage, Mad City Labs) was used to control the two-dimensional position of the flow cell in optical trapping experiments. A single-axis piezo-*z*-scanning stage (Nano-Z100, Mad City Labs) was used for 3D imaging of gold and/or fluorescence microspheres during system alignment. The fluorescence signal from the sample was collected by the same objective, descanned by the scanner and then separated from the excitation beam using a dichroic mirror (Di03-R405/488/532/635-t3-25×36, Semrock). Two further dichroic mirrors (FF560-FDi01-25×36 and FF640-FDi01-25×36, Semrock) were used to split the fluorescence signal into three separate colour channels. The photons were additionally filtered using appropriate emission filters (blue: FF03-525/50-25 and NF01-532U-25, Semrock; green: FF01-582/75-25, Semrock; red: F42-652 and F47-686, AHF) and then coupled into a fibre-coupled avalanche photodiode (APD; SPCM-AQRH-14-FC; fibre, SPCM-QC9; PerkinElmer) using achromatic lenses (blue: AC254-150-A; green: AC254-125-A; red: AC254-100-A; Thorlabs), where the multimode fibre (50-µm diameter, ~1.75 Airy discs) served as the confocal pinhole. A short-pass filter (F75-720, AHF) was used before the APDs to block the remaining back reflections of STED light.

For the imaging of gold beads during the alignment, the optical path was split by introducing a pellicle beamsplitter (BP145B1, Thorlabs) on a magnetic holder just before the scanner. Using an achromatic lens (AC254-050-AB, Thorlabs), the light was coupled into a fibre-coupled APD (SPCM-AQRH-14-FC; fibre, FG200UEA; Thorlabs) with a larger collection angle than the imaging APDs (200-µm-diameter pinhole, >10 Airy discs).

### Optical trapping and axial imaging of optically trapped samples

**Extended Data Fig. 5:**
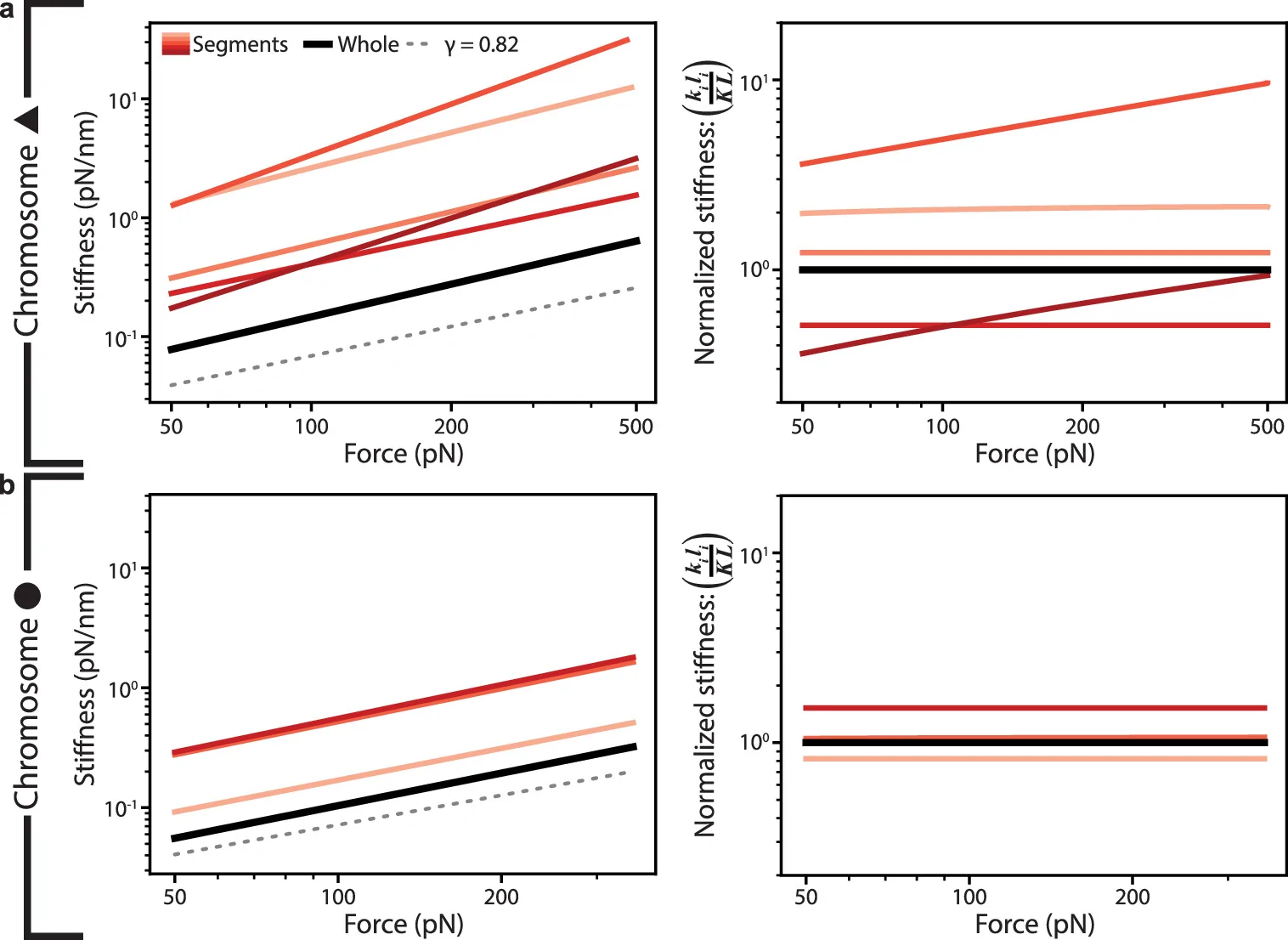
Stiffness-force curves of selected chromosomes. **a** & **b**: Stiffness-force curves, calculated using the generalized WLC formula fits, for the segments and the whole of the ▲-chromosome and ●-chromosome as in Fig. 3. The figure on the top left is identical to Fig. 4i but is reproduced here for convenience. On the left, the stiffness is plotted. In this form, the stiffness of the segments is always higher than the stiffness of the total chromosome, and the data is not corrected for variations in segment length. On the right, we plotted the normalized stiffness (Methods). Here, any number above 1 means that the stiffness is higher than would be expected in a homogeneous chromosome, and any number below 1 that the stiffness is lower. Positive slopes mean that the segment stiffens faster than the total chromosome, and negative slopes means that it stiffens slower. The ▲-chromosome was segmented into 5 parts, which show a wide range of stiffnesses and normalized stiffnesses. The ●-chromosome was segmented into only 3 parts, which are all relatively similar to the total chromosome.

**Extended Data Fig. 6:**
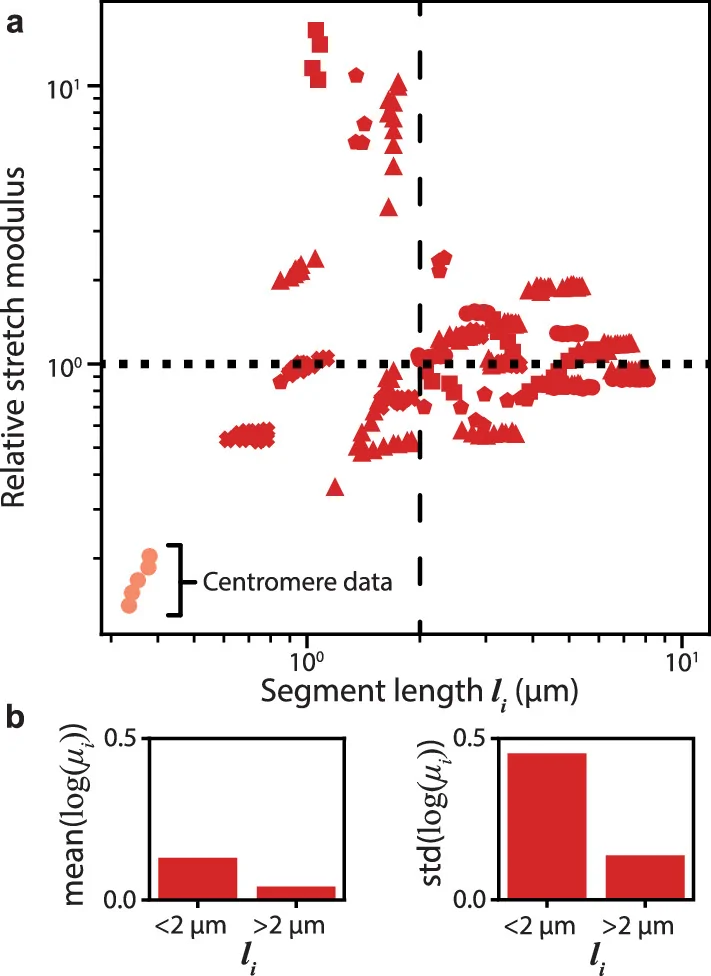
Relative stretch modulus vs. absolute segment length. **a**: Relative stretch moduli as in Fig. 4g in the main text, plotted against the absolute segment length l_i_, instead of the relative segment length *l*_*i*_/*L*. Here we don’t indicate the inaccessible region, as in Fig. 4g, because the absolute length scale of the inaccessible region depends on the whole chromosome length *L* and is different for each chromosome. **b**: Left: mean of the logarithm of the relative stretch modulus, for data from A. Statistical testing (two-sided Welch’s t-test, p = 0.336) suggest no significant difference between the means of the two groups. Right: standard deviation of the logarithm of the relative stretch modulus, for data from A. Levene’s test of equal variance (two-sided) confirms that the difference in the variance between groups is statistically significant (p = 4.6e-17).

Optical trapping was performed using a 20-W continuous-wave fibre laser (YLR-20-LP, 1,064 nm, IPG Photonics), similar to what was described previously^6^. The laser beam was expanded using a 4*f* telescope system with 75-mm- and 150-mm-focal-length lenses. The 75-mm lens was mounted on an automated linear stage (AG-LS25, Newport) to control the collimation of the trapping beam for *z* scanning in confocal and STED imaging of the optically stretched samples. Two polarizing beamsplitter cubes (10BC16PC.9, Newport) were used to first split and then recombine the 1,064-nm laser for obtaining two independently steerable optical traps. One accurate piezo-controlled mirror (Nano-MTA2X Aluminum, Mad City Labs) and another coarse-positioning steering mirror (AG-M100N, Newport) were used for the beam steering of the two traps. The trapping beams were then coupled into the objective by a 4*f* telescope system using two 300-mm lenses. Transmitted and scattered trapping beams were collected using the condenser lens (P 1.40 OIL S1 11551004, Leica) and then coupled onto two position-sensitive detectors (DL100-7PCBA3, Pacific Silicon Sensor) after separation of the two orthogonally polarized beams using a polarizing beamsplitter cube. The force detection was performed by measuring and converting the displacements of the scattered trapping beams, using the basic principle of back-focal-plane interferometry^59^. Wide-field imaging of the microspheres and optically stretched samples was performed using a complementary metal–oxide–semiconductor camera (DCC1545M, Thorlabs) and transmitted 875-nm LED illumination (LED-1115-ELC-875-195, IMM Photonics). The LED illumination was separated from the trapping light using two dichroic mirrors before and after the flow cell. The power of the 1,064-nm laser was tuned to obtain approximately 3.5 W per trap for the DNA experiments (Fig. 1e and Extended Data Fig. 1c) and 5 W per trap for chromosome experiments (this number corresponds to the laser power that is coupled in to the system; the laser power that reaches the sample can be tuned using a combination of rotating wave plates and polarizing beamsplitters). For 4.47-µm microspheres, these powers yield a trap stiffness of roughly 0.4 pN nm^−1^ and 0.6 pN nm^−1^, and allows stretching forces up to 400 pN and 600 pN, respectively.

### Spatial phase modulation and sensorless AO using SLM

Spatial phase modulation and sensorless AO were performed using the SLM in the 766-nm laser path to generate the desired STED focal intensity distribution in the sample even in the presence of system-induced optical aberrations. The laser beam was sequentially modulated first by a (Extended Data Fig. 2a) and then by an (Extended Data Fig. 2b) phase mask in a polarization multiplexing manner. To do this, the expanded 766-nm laser beam was first guided to the left part of the SLM liquid-crystal screen, and then a 90° turn of all the polarization components was applied to the beam, and it was imaged back onto the right part of the screen using a 4*f* telescope system with a quarter-wave plate. Hence, the two orthogonal linearly polarized components of the 766-nm laser were phase modulated independently by the helical and annular phase masks on the SLM. In the sample, the resulting focal intensity distribution exhibits an incoherent superposition of the doughnut- and bottle-shaped point spread functions that enable lateral and axial STED. The system can then work in the lateral mode, axial mode or a combination of two modes with a tunable imaging resolution along all three spatial dimensions, by using different orientations of the half-wave plate at the couple-in side of the STED laser. To minimize the negative influence of the imperfect phase modulation efficiency of the SLM on the STED performance, the undesired unmodulated light of the 766-nm laser was separated from the desired modulated light using an additional holographic grating in the phase masks. The size of each phase mask (570 × 570 pixel^2^, approximately 7-mm diameter) was controlled to adapt to the beam size on the SLM. A flatness-correction phase mask that was obtained using an interferometric method was applied to the SLM to minimize the wavefront distortion caused by manufacturing errors in the liquid-crystal panel.

Sensorless AO was performed to detect the optical aberrations that were present in the 766-nm STED path and to compensate for them using the SLM. To this end, aberrations were described as a linear combination of the first 15 modes of the Zernike polynomials (except the modes corresponding to piston, tip, tilt and defocus (Extended Data Fig. 2d), and AO was used to find the amplitudes of the modes. In practice, the confocal PSFs of the STED path (without any helical or annular phase mask) were imaged using gold microspheres and evaluated using appropriate metrics (sum intensity, sum-squared intensity and image sharpness). Then, the PSF was iteratively improved by optimizing the chosen metric and varying the amplitude of each of the Zernike modes in succession. Finally, using the determined amplitudes, an AO-compensation phase mask could be generated and then applied to the STED helical and/or annular phase masks on the SLM. The AO procedure can be performed for multiple iterations to improve the accuracy of the final results.

The phase distribution of Zernike polynomials, the STED masks and holographic grating were pregenerated using a custom (version R2019a)^60^ program and stored on the computer to improve the subsequent calculation efficiency. The STED helical and annular phase distribution, holographic grating and phase mask for SLM flatness correction were combined with a specific AO-compensation mask, phase wrapped in the range of 0–2π and sent to the SLM using a LabVIEW (2011) program. The phase modulation accuracy was improved (compared with the 8-bit mode, 256-level phase modulation) using the 12-bit mode (4,096-level phase modulation) of the SLM, where all the generated phase distributions (grey-level images) were converted into RGB images. Specifically, for each pixel in the generated phase distribution, the value (1,000, for example) was first divided by 256, and then the quotient (3; 0011 in 4-bit notation) and remainder (232; 11101000 in 8-bit notation) were assigned to a lower 4-bit green channel and an 8-bit blue channel of the RGB image, respectively.

### Hardware control and data acquisition

For force detection, the output signals from the PSDs were sampled at 50 kHz using a data acquisition card (NI PCI-4472B, National Instruments). A multifunction card (NI PCIe-6323, National Instruments) was used for confocal scanning, photon counting and digital input/output. The optical trapping, confocal and STED imaging were controlled using custom-built LabView (2011) software.

### Microfluidics, DNA, proteins and sample preparation

A five-channel laminar flow cell (LUMICKS) was mounted on an automated *x*–*y* stage (MS-2000, Applied Scientific Instrumentation), which allowed the rapid transfer of DNA- and chromosome-dumbbell constructs between different flow channels.

Bacteriophage λ-DNA samples were prepared and biotinylated as described previously^61^. Streptavidin-coated microspheres (Spherotech) were used for the optical trapping of DNA and chromosomes.

Here 20 nM of interacalator (Yopro-1, Invitrogen) was used for the 3D confocal imaging of optically stretched multiple DNA-dumbbell construct in a phosphate-buffered saline buffer (Extended Data Fig. 1c).

The fluorescently labelled BsoBI-Atto647N and EcoRV-Atto647N restriction enzymes were prepared as described previously^62^. The DNA–protein complexes were obtained by preincubating 10 nM of restriction enzymes with biotinylated λ-DNA in Tris buffer (containing 10 mM of Tris at pH 7.5, 20 mM of NaCl, 2.5 mM of CaCl_2_, 0.5 mM of EDTA, 0.02% Pluronic and 0.02% bovine serum albumin) for approximately 30 min at room temperature (Fig. 1e).

For the alignment of the confocal and STED microscope, a 3D sample was prepared by mixing 50-nm-diameter gold beads (1:20 v/v, gold colloid 50 nm, EM. GC50, BBInternational), 20-nm-diameter crimson fluorescent beads (1:2,000 v/v, 625/645, F8782, Invitrogen) and agarose (1:40 w/v, BP160-500, Fisher BioReagents) with water. The full protocol for producing gold bead and fluorescent bead samples was published before^63^.

### Chromosomes and (sparse) immunofluorescence staining

U2OS cells (cell line^21^) were grown in the laboratory and the mitotic chromosomes were isolated and site-specifically biotinylated to bind them to microspheres for optical trapping, as described previously^21,22^. PA buffer (containing 15 mM of Tris-HCl at pH 8, 2 mM of EDTA, 80 mM of KCl, 20 mM of NaCl, 0.5 mM of EGTA, 0.5 mM of spermidine, 0.2 mM of spermine and 0.2% Tween-20) was used during chromosome isolation and optical tweezers experiments.

Following a conventional immunofluorescence staining protocol, the mitotic chromosomes were incubated overnight at 4 °C with the appropriate primary antibody—anti-CENP-A (1:100, produced in mouse, ab13939, Abcam, lot: GR3259853-3, discontinued), anti-NCAPH (1:100, produced in rabbit, HPA002647, Sigma, lot: A81606)—after diluting using PA buffer with 2% bovine serum albumin (heat-shock fraction, A7030-10G, Sigma) buffer. The samples were diluted by a factor of 4 and then centrifuged at 750*g* for 5 min with glycerol at the bottom of the tube to remove the free antibody. The suspension was then removed carefully. A second dilution was performed before an appropriate secondary antibody—anti-mouse-IgG-Atto647N (1:200, 50185-1ML-F, Sigma) and anti-rabbit-IgG-Atto647N (1:200, 40839-1ML-F, Sigma)—was added. The samples were centrifuged again after incubation with the secondary antibody for at least 1 h at room temperature.

Alternatively, for more direct control over the sparsity of the staining of trapped chromosomes, a side channel of the flow cell was loaded with ~5 nM of fluorescently labelled anti-NCAPG (produced in mouse, H00064151-M01, Abnova, lot: IB281-4B1). Similar to NCAPH, NCAPG is a component of the condensin I protein complex. A fluorescent label was conjugated to the primary antibodies directly using an Alexa 647 NHS ester. The trapped chromosomes were stained by moving them to the side channel containing the antibodies, and the density of staining could be tuned by altering the duration of the incubation there. To prevent photobleaching, a mixture of glucose oxidase, catalase^64^, glucose and Trolox^65^ was added to the experimental buffer for these experiments.

### Chromosome-stretching experiments and visual segmentation

Chromosome-stretching experiments were largely handled as described before^21,22^. After catching (and antibody incubation), the sparsely stained chromosomes was sequentially force clamped at increasing forces (50–500 pN). The force clamp increased the force by 50–100 pN between measurements, at a rate of ~400 pN s^−1^ between measurements. After each force increase, the chromosome was held for 5 s to allow for the elastic contribution of the extension to stabilize, before it was confocally imaged. Thus, the effective loading rate was 10 pN s^−1^. These experiments resulted in a force stack in which the sparsely labelled loci can be tracked through all images.

We used ImageJ plugin TrackMate^66,67^ (v. 7.14.0) to track the loci throughout the force stack, making sure to only select loci close to the chromosome axis, and loci that are present in all images of the stack. The edges of the chromosome could be determined by using the H2B-EGFP fluorescence signal or, if that was not imaged, the chromosomal autofluorescence background signal.

We projected the loci on the chromosome’s long axis, effectively only considering their coordinates along the chromosome’s length. Segment lengths were then defined as the distances between pairs of loci.

To calculate the error bars, we assumed the localization accuracy of the locus tracking to be $\Delta\,=$ 25 nm, or equivalent to half a pixel. This value is relatively high^68^, but we believe it to be a reasonable estimate considering the fact that a fraction of loci will always be slightly defocused in the thick chromosome sample.

### Apparent stiffness and normalization

**Extended Data Fig. 7:**
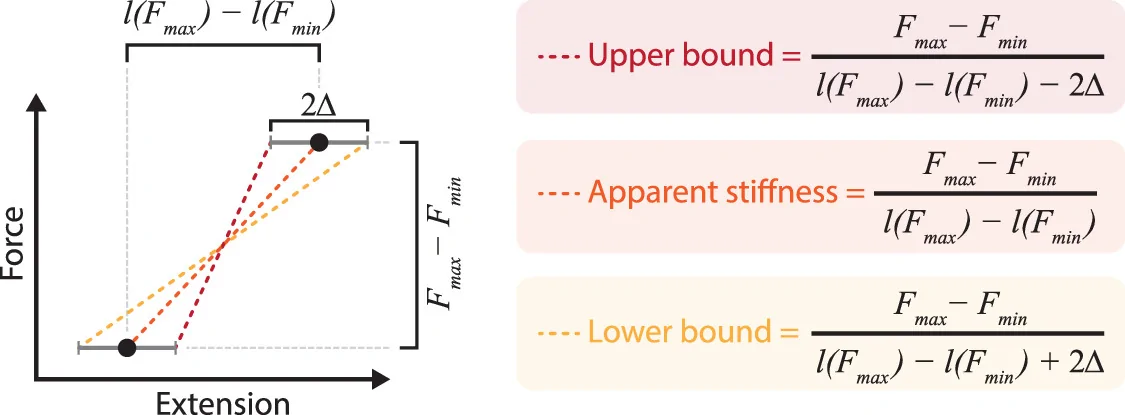
Calculating upper and lower bounds for the apparent stiffness. Schematic explaining how upper (*k*_upp_) and lower (*k*_low_) bounds of the apparent stiffness (*k*) are determined. The extension l of a segment is measured at *F*_max_ and *F*_min_. Since the extension is measured by tracking the distance between two fluorescence spots, the error on the extension is determined by the localization precision Δ of the microscope. Upper and lower bounds are calculated by considering this localization precision, as indicated in the figure. Since the localization precision is independent of the segment stiffness, the errors are generally higher for stiffer segments with smaller extensions. Another effect of this method of error calculation, is that error bars are asymmetric (see Figs. 2 and 3). Considering the equations above it is straightforward to see that $\left|{k}_{\rm{upp}}-k\right| > \left|{k}_{\rm{low}}-k\right|$ holds for any segment. For very stiff segments, $l\left({F}_{\max }\right)-l({F}_{\min })\le 2\Delta$ might hold: the extension is smaller than the localization precision. In these cases, ${k}_{\rm{upp}}$ goes to infinity. Nevertheless, determining ${k}_{\rm{low}}$ is still possible.

The apparent stiffness for all segments and chromosomes was determined by calculating

5 $${k}_{i}=\frac{{F}_{\max }-{F}_{\min }}{{l}_{i}\left({F}_{\max }\right)-{l}_{i}\left({F}_{\min }\right)}$$

where *F* is the force and ${l}_{i}(F)$ is the length of segment *i* at force *F*. The lower bound for the apparent stiffness was calculated as

6 $${k}_{\mathrm{lower},i}=\frac{{F}_{\max }-{F}_{\min }}{{l}_{i}\left({F}_{\max }\right)-{l}_{i}\left({F}_{\min }\right)+2{\Delta}}$$

and the upper bound as

7 $${k}_{{\mathrm{upper}},i}=\frac{{F}_{\max}-{F}_{\min }}{{l}_{i}\left({F}_{\max }\right)-{l}_{i}\left({F}_{\min }\right)-2\Delta},$$

where Δ again is the localization precision. Extended Data Fig. 7 schematically illustrates the procedure for the calculation of these bounds. For stiff segments, the localization precision is relatively large compared with their extension; therefore, the errors for segments with high apparent stiffnesses are generally larger (Fig. 3e). Also, the error bars are asymmetric: from equations (5)–(7), it follows that $\left|{k}_{\rm{upper}}-k\right| > \left|{k}_{\rm{lower}}-k\right|$ always holds. For some of the stiffer segments, the upper limit on the stiffness goes to infinity, because the extension curve is so steep that the localization precision is larger than the segment’s extension. For these segments, the lower limit on the stiffness is the more interesting value to consider.

Since the stiffness of a segment of the chromosome is expected to vary with the segment length, we formulate a null hypothesis for the expected stiffnesses of chromosome segments if there were no spatial heterogeneity. Consider a chromosome consisting of N segments in series. At any given force F, the instantaneous compliances (inverses of the stiffnesses ${k}_{i}$) of the serial elements add:

8 $$\frac{1}{K(F)}=\mathop{\sum }\limits_{i=1}^{N}\frac{1}{{k}_{i}(F)}.$$

If the chromosome were a homogeneous material, this would imply that the compliance of a segment would be proportional to its length: $\frac{1}{{k}_{i}}=\frac{{l}_{i}}{{LK}}$. We, therefore, define the relative stretch modulus of an element as ${\mu }_{i}={k}_{i}{l}_{i}/\left({LK}\right)$, and interpret the relative stretch modulus values below or above one as signs of spatial heterogeneity across the length of the chromosome.

Since the errors on ${k}_{i}$ and K are asymmetric, standard error propagation cannot be used to determine the error on ${\mu }_{i}.\,$Instead, we calculated the error bars for the relative stretch modulus ${{\mu }_{i}=k}_{i}{l}_{i}/\left({LK}\right)$ by determining a lower and upper bound. The lower bound is given by

$${\mu }_{\mathrm{lower},i}=\frac{{k}_{\mathrm{lower}}\times ({l}_{i}-\Delta)}{{K}_{\mathrm{upper}}\times (L+\Delta)\,},$$

and the upper bound is given by

$${\mu }_{{\rm{upper}},i}=\frac{{k}_{\rm{upper}}\times ({l}_{i}+\Delta)}{{K}_{\rm{lower}}\times (L-\Delta)\,}.$$

### Fitting force–extension data using the Marko–Siggia WLC model

We used a previously published approximated inversion^69^ of the Marko–Siggia WLC formula to fit our data to the conventional WLC model.

### Fitting force–extension data for abnormal stiffening exponents

Equations (3) and (4) can be used to approximate the force–extension behaviour with an asymptotic stiffening exponent of $0 < {{\gamma }} < 1$ or ${{\gamma }} > 1$. We also note that an expression for the limiting case of ${{\gamma }}=1$ can be written as

9 $$\frac{F}{{f}_{\mathrm{c}}}\approx \frac{l}{{l}_{\mathrm{c}}}+{B}_{1}\left({e}^{\frac{l}{{l}_{\mathrm{c}}}}-1\right)$$

where the positive constant of order unity, ${B}_{1}$, is now set by ${k}_{0}=\left(1+{B}_{1}\right)\frac{f_{\mathrm{c}}}{l_{\mathrm{c}}}$.

We now wish to consider how to best fit the experimental data for the extensions of a chromosome segment, without prior knowledge of the stiffening exponent that describes the segment’s high-force response. For simplicity, we consider exponents ${{\gamma }}\ne 1$. Since our experimental force range does not include the linear-response regime, we cannot resolve ${k}_{0}$ or ${f}_{\mathrm{c}}$. Given this consideration, in equation (4), there are effectively only two unknown prefactors:

10 $${F}\approx {A}^{{\prime} }l+{B}^{{\prime} }{l}^{1/\left(1-{{\gamma }}\right)}$$

where $${A}^{{\prime} }={A}_{ < }{f}_{\mathrm{c}}/{l}_{\mathrm{c}}$$ and $${B}^{{\prime} }={B}_{ < }{f}_{\mathrm{c}}{l}_{\mathrm{c}}^{-1/\left(1-{{\gamma }}\right)}$$.

In equation (3), we fix ${B}_{ > }=1/4$ for consistency with flexible WLC behaviour (equation (2)). We note that in the high-force regime, the linear term gives a negligible contribution; therefore, a different value of ${B}_{ > }$ is equivalent to a rescaling of ${f}_{\mathrm{c}}$:

11 $$\frac{F}{{f}_{\mathrm{c}}^{{\prime} }}\approx \frac{1}{4}\left({\left(1-\frac{l}{{l}_{\mathrm{c}}}\right)}^{1/\left(1-{{\gamma }}\right)}-1\right)\approx \frac{{\rm{l}}}{l_{\mathrm{c}}}+\frac{1}{4}\left({\left(1-\frac{l}{{l}_{\mathrm{c}}}\right)}^{1/\left(1-\gamma \right)}-1\right)$$

where $${f}_{\mathrm{c}}^{{\prime} }={4B}_{ > }{f}_{\mathrm{c}}$$.

We, hence, first separately fit equations (10) and (11) to the force–extension data for a single chromosome segment, varying γ in the appropriate ranges. We then compare the fit residues to determine which stiffening exponent γ best describes the nonlinear force response of the chromosome segment.

Both equations (10) and (11) are of the form $F\left(l\right)$ and are non-invertible, whereas our data are more accurately fitted by a function of the form $l\left(F\right)$, since the measurement errors in the force are very small compared with the measurement errors in the distance. To overcome this problem, in each iteration of our fitting algorithm, we used equations (10) and (11) to calculate *F*(*l*, fit parameters) for a range of tightly meshed *l* values, and then used linear interpolation to numerically determine *l*(*F*, fit parameters) for comparison with the *l*(*F*) data.

### Reporting summary

Further information on research design is available in the Nature Portfolio Reporting Summary linked to this article.

## Online content

Any methods, additional references, Nature Portfolio reporting summaries, source data, extended data, supplementary information, acknowledgements, peer review information; details of author contributions and competing interests; and statements of data and code availability are available at https://doi.org/10.1038/s41567-026-03407-z.

## Supplementary information

Supplementary Information Supplementary Notes 1 and 2.

Reporting Summary

## Data Availability

The data supporting the findings in this study are available via Dataverse at https://doi.org/10.34894/WC88VR (ref. ^70^).

## Code availability

The Python 3 analysis code is available via Dataverse at https://doi.org/10.34894/WC88VR (ref. ^70^).
