# Supplementary material for: Large intra-chromosome heterogeneity leads to emergent nonlinear mechanics
Source: Nat Phys. 2026 Aug 7;22(10):1655–62. doi: 10.1038/s41567-026-03407-z (PMC13658011; doi:10.1038/s41567-026-03407-z)
Supplement: Supplementary file 1 [file 41567_2026_3407_MOESM1_ESM.pdf]

nature physics

Supplementary information https://doi.org/10.1038/s41567-026-03407-z

Large intra-chromosome heterogeneity leads to emergent nonlinear mechanics

In the format provided by the authors and unedited

Supplementary information

Supplementary note 1: additional information on adaptive optics (AO)

We used the phase modulation capabilities of the SLM for sensorless adaptive correction of aberrations (or: Adaptive Optics, AO) in the 766 nm STED laser pathway. We based our AO procedure on the expansion of aberrations into the first 15 modes of the Zernike polynomials [1,2], excluding modes corresponding to piston, tilt, and defocus, because these shift the location of the focus and are easier to correct for manually (Fig. S2D) [3–5]. By linearly combining the Zernike modes with the STED phase masks on the SLM, each mode can be used to correct for a specific type of aberration and this correction can be optimized by tuning the amplitude of the mode to maximize some predefined merit function. The Zernike modes are orthogonal in the pupil plane, and we thus found we can successfully optimize the modes in an independent, sequential manner.

To perform our automated AO procedure we first removed the microfluidic flow cell, and replaced it with a piezoelectrically operated z-stage containing a sample of 50 nm gold nanoparticles in an agarose gel. By inserting a pellicle beamsplitter into the STED pathway (Fig. S1A), we could use the reflection off the gold nanoparticles to image the PSF of the STED beam in xy and xz (using the z-stage). We chose to optimize the STED PSF without any helix or annular phase mask, and simply defined the merit function as the sum of the squared pixel intensities. Then, iterating over the Zernike modes, the effect on the merit of each mode was tested over a range of mode amplitudes, and by fitting a Gaussian curve to these measurements, the optimal value for each mode's amplitude was determined (Figs. S2E-G). During the AO procedure, the merit increased overall, and for most individual modes, but for some modes we saw a decrease in merit after optimization. This can occur either because of sample drift/defocusing, or because of a poor fit to the 5 data points. The former is not problematic, because the individual mode amplitudes will still be optimized correctly; the latter can be easily (manually) corrected without the need to rerun the entire procedure, since the Zernike modes are orthogonal. The final phase mask to be projected on the SLM is a linear combination of the flatness correction map as provided by the SLM manufacturer, the helix and annular phase masks, a holographic grating to filter out non-modulated light, and the Zernike modes at the thus determined amplitudes (Fig S2H). The aberration compensation phase mask, that was optimized using the unmodulated STED beam, significantly improves the quality of both the lateral and axial STED PSFs when it comes to symmetry, brightness of the ring, and darkness of the center (Fig. S2C).

Supplementary note 2: centromere softness and spindle attachment correction

When a cell divides, it needs to evenly distribute the two copies of its genome over both daughter cells. To achieve this, the cell assembles a microtubule structure—the mitotic spindle—at each cell pole. The mitotic spindle attaches to the kinetochores of each mitotic chromosome, located on both sister chromatids at the outer centromere, and then pulls the chromatids toward opposite poles. For proper segregation, it is hence essential that the two kinetochores of each mitotic chromosome are connected to spindle fibers from opposite poles.

It was theorized that the cell can distinguish correct (amphitelic) spindle attachment from erroneous (syntelic, merotelic) attachment through a tension sensing mechanism involving Aurora B Kinase[6–8]. Aurora B Kinase promotes the turnover of spindle-kinetochore attachments through the phosphorylation of kinetochore substrates, and it is enriched in the inner centromere during metaphase. When the kinetochores are correctly attached to the respective poles, a tension is applied between them. Thus, the correctly attached spindle pulls both kinetochores apart and spatially separates them from the Aurora B Kinase reservoir in the inner centromere. As a result, Aurora B Kinase cannot detach the kinetochores from the spindle, and attachment is retained until the end of anaphase. Conversely, in an erroneous spindle configuration, connecting both sister chromatids to the same pole, no tension is applied between kinetochores. Consequently, the kinetochores remain adjacent to the inner centromere, and Aurora B Kinase will eventually release the spindle-kinetochore attachment.

In the described system, a softer centromere, as found in our experiments, would result in a more sensitive sensor for spindle attachment, because any applied tension would lead to a larger spatial separation of the kinetochores. Accordingly, the sensor would be able to resolve more subtle deviations in tension, and thus correct for more subtle errors, like the merotelic configuration in which one kinetochore is attached to both poles. Our finding that the centromere is exceptionally soft may hence be related to its function as a tension sensor during mitosis. However, it should be noted that we probed the centromere stiffness along the chromosome's long axis, while the forces exerted by the mitotic spindle are directed perpendicular to the long axis. Further experiments, using optical tweezers or other techniques like AFM, are needed to probe the centromere stiffness in the perpendicular direction.

Supplementary references

1. Zernike, V. F. Beugungstheorie des schneidenver-fahrens und seiner verbesserten form, der phasenkontrastmethode. *Physica* **1**, 689–704 (1934).
2. McAlinden, C., McCartney, M. & Moore, J. Mathematics of Zernike polynomials: a review. *Clin. Experiment. Ophthalmol.* **39**, 820–827 (2011).
3. Love, G. D. Wave-front correction and production of Zernike modes with a liquid-crystal spatial light modulator. *Appl. Opt.* **36**, 1517–1524 (1997).
4. Gould, T. J., Burke, D., Bewersdorf, J. & Booth, M. J. Adaptive optics enables 3D STED microscopy in aberrating specimens. *Opt. Express* **20**, 20998 (2012).
5. Hampson, K. M., Antonello, J., Lane, R. & Booth, M. Sensorless Adaptive Optics. https://doi.org/10.5281/zenodo.4066425 (2020) doi:10.5281/zenodo.4066425.
6. Lampson, M. A. & Grishchuk, E. L. Mechanisms to Avoid and Correct Erroneous Kinetochore-Microtubule Attachments. *Biology* **6**, 1 (2017).
7. Andrews, P. D. *et al.* Aurora B Regulates MCAK at the Mitotic Centromere. *Dev. Cell* **6**, 253–268 (2004).
8. Zaytsev, A. V. *et al.* Bistability of a coupled Aurora B kinase-phosphatase system in cell division. *eLife* **5**, e10644 (2016).
