# Supplementary material for: Large intra-chromosome heterogeneity leads to emergent nonlinear mechanics
Source: Nat Phys. 2026 Aug 7;22(10):1655–62. doi: 10.1038/s41567-026-03407-z (PMC13658011; doi:10.1038/s41567-026-03407-z)
Supplement: Supplementary file 2 [file 41567_2026_3407_MOESM2_ESM.pdf]

nature portfolio

Corresponding author(s): Erwin J. G. Peterman
Gijs J. L. Wuite
Chase P. Broedersz

Last updated by author(s): Jun 19, 2026

Reporting Summary

Nature Portfolio wishes to improve the reproducibility of the work that we publish. This form provides structure for consistency and transparency in reporting. For further information on Nature Portfolio policies, see our Editorial Policies and the Editorial Policy Checklist.

Statistics

For all statistical analyses, confirm that the following items are present in the figure legend, table legend, main text, or Methods section.

n/a	Confirmed	
☐	☒	The exact sample size (*n*) for each experimental group/condition, given as a discrete number and unit of measurement
☐	☒	A statement on whether measurements were taken from distinct samples or whether the same sample was measured repeatedly
☐	☒	The statistical test(s) used AND whether they are one- or two-sided<br>*Only common tests should be described solely by name; describe more complex techniques in the Methods section.*
☒	☐	A description of all covariates tested
☒	☐	A description of any assumptions or corrections, such as tests of normality and adjustment for multiple comparisons
☐	☒	A full description of the statistical parameters including central tendency (e.g. means) or other basic estimates (e.g. regression coefficient) AND variation (e.g. standard deviation) or associated estimates of uncertainty (e.g. confidence intervals)
☐	☒	For null hypothesis testing, the test statistic (e.g. *F*, *t*, *r*) with confidence intervals, effect sizes, degrees of freedom and *P* value noted<br>*Give P values as exact values whenever suitable.*
☒	☐	For Bayesian analysis, information on the choice of priors and Markov chain Monte Carlo settings
☒	☐	For hierarchical and complex designs, identification of the appropriate level for tests and full reporting of outcomes
☒	☐	Estimates of effect sizes (e.g. Cohen's *d*, Pearson's *r*), indicating how they were calculated

Our web collection on statistics for biologists contains articles on many of the points above.

Software and code

Policy information about availability of computer code

Data collection	Custom made LabVIEW (2011) software was used to obtain optical tweezers data.
Data analysis	FIJI / ImageJ was used for the analysis of images, specifically the plugin TrackMate (v.7.14.0). Custom made Python 3 (3.11.15) scripts were used to further analyze data. Custom made Julia (1.10) scripts were used to generate figures 4A, 4B, and the inset in 4E.

For manuscripts utilizing custom algorithms or software that are central to the research but not yet described in published literature, software must be made available to editors and reviewers. We strongly encourage code deposition in a community repository (e.g. GitHub). See the Nature Portfolio guidelines for submitting code & software for further information.

Data

Policy information about availability of data

All manuscripts must include a data availability statement. This statement should provide the following information, where applicable:

- Accession codes, unique identifiers, or web links for publicly available datasets
- A description of any restrictions on data availability
- For clinical datasets or third party data, please ensure that the statement adheres to our policy

The data and data analysis code files supporting the findings in this study are openly available from the DataverseNL repository at https://doi.org/10.34894/WC88VR

Research involving human participants, their data, or biological material

Policy information about studies with human participants or human data. See also policy information about sex, gender (identity/presentation), and sexual orientation and race, ethnicity and racism.

Reporting on sex and gender	This study involves no human participants or data.
Reporting on race, ethnicity, or other socially relevant groupings	This study involves no human participants or data.
Population characteristics	This study involves no human participants or data.
Recruitment	This study involves no human participants or data.
Ethics oversight	No external ethical approval was necessary for the experiments in this study.

Note that full information on the approval of the study protocol must also be provided in the manuscript.

Field-specific reporting

Please select the one below that is the best fit for your research. If you are not sure, read the appropriate sections before making your selection.

☒ Life sciences ☐ Behavioural & social sciences ☐ Ecological, evolutionary & environmental sciences

For a reference copy of the document with all sections, see nature.com/documents/nr-reporting-summary-flat.pdf

Life sciences study design

All studies must disclose on these points even when the disclosure is negative.

Sample size	No sample size calculations were performed. The sample sizes are indicated in the figure captions.<br>For our low-throughput experiments, sample sizes were based on feasibility within time constraints. The sample size of 5 visually segmented chromosomes is large enough to show significant variation in segment stiffness. The sample size for our experiments on centromeres is small, but our findings are supported by previous work (Sacristan, 2024).
Data exclusions	Data was excluded when the full experiment could not be carried out before rupture or detachment of the chromosomes. Data was excluded when chromosome segment lengths could not be accurately determined for the full force sweep because of drift or photobleaching.
Replication	Our findings on visually segmented chromosomes were replicated, and all our data is available in the manuscript. Experiments were carried out by different people, on different days, and on different chromosome isolations. Total sample size is 5.<br>STED images of chromosomes with immunostained centromeres were routinely obtained. They rendered high-quality quantifiable data in one instance. These are presented in the manuscript, and are in good agreement with earlier experiments (Sacristan, 2024), as discussed in the main text. In this one instance, we observed a clear bipartite structure for one of two centromeres. We did not see the bipartite structure more often, likely because of the very small size of the respective parts, skewed orientation of chromosomes relative to the xy-plane, and focus drift.
Randomization	Chromosomes are randomly selected and tethered by the process described in the Methods section. We cannot select for specific chromosomes, but are probably biased towards larger chromosomes because they are easier to spot through the microscope. We see no reason to expect this has affected our conclusions.
Blinding	In our study there was only one allocated group (just wild types and no controls) so blinding was not necessary.

Reporting for specific materials, systems and methods

We require information from authors about some types of materials, experimental systems and methods used in many studies. Here, indicate whether each material, system or method listed is relevant to your study. If you are not sure if a list item applies to your research, read the appropriate section before selecting a response.

Materials & experimental systems

n/a	Involved in the study
☐	☒ Antibodies
☐	☒ Eukaryotic cell lines
☒	☐ Palaeontology and archaeology
☒	☐ Animals and other organisms
☒	☐ Clinical data
☒	☐ Dual use research of concern
☒	☐ Plants

Methods

n/a	Involved in the study
☒	☐ ChIP-seq
☒	☐ Flow cytometry
☒	☐ MRI-based neuroimaging

Antibodies

Antibodies used

Primary antibodies:
Anti-CENP-A (1:100, Abcam, ab13939, produced in mouse, monoclonal, lot: GR3259853-3, discontinued)
Anti-NCAPH (1:100, Sigma, HPA002647, produced in rabbit, polyclonal, lot: A81606)

Secondary antibodies:
Anti-Mouse-IgG-Atto647N (1:200, Sigma, 50185-1ML-F)
Anti-Rabbit-IgG-Atto647N (1:200, Sigma, 40839-1ML-F)

Used in solution for sparse staining:
anti-NCAPG (~5 nM, Abnova, H00064151-M01, produced in mouse, monoclonal, lot: IB281-4B1)

Validation

ab13939 (CENP-A) was validated by WB and ICC, and was used in Ly et al. 2019, Nature Genetics, and 183 other publications. The antibody was discontinued by the manufacturer because its production - using the ascites method - does not comply with current animal welfare standards.

HPA002647 (NCAPH) was validated by IHC and WB, and was used in the Human Protein Atlas and in Zhan et al. 2018, Oncol Rep, and Scott et al. 2017, Mol Syst Biol.

H00064151-M01 (NCAPG) was used in Satow (2010) and validated by the manufacturer.

Eukaryotic cell lines

Policy information about cell lines and Sex and Gender in Research

Cell line source(s)

The U2OS cell line was previously published in Meijering et al. 2022.
They indicate that: the parental U2OS cells were obtained from and authenticated by ATCC (USA). They were modified into U2OS BirA-TRF1 cells and authenticated like described in Garcia-Exposito et al. 2016, Cell reports.

Authentication

The ATCC authenticated the wild type U2OS cell line used in this study by STR profiling.

Mycoplasma contamination

All cell lines were tested negative for mycoplasma contamination.

Commonly misidentified lines
(See ICLAC register)

No commonly misidentified cell line was used in this study.

Plants

Seed stocks

This study involves no plants.

Novel plant genotypes

This study involves no plants.

Authentication

This study involves no plants.
